# Microfluidic impedance flow cytometer leveraging virtual constriction microchannel and its application in leukocyte differential

**DOI:** 10.1038/s41378-024-00833-y

**Published:** 2024-12-16

**Authors:** Minruihong Wang, Jie Zhang, Xiao Chen, Yimin Li, Xukun Huang, Junbo Wang, Yueying Li, Xiaoye Huo, Jian Chen

**Affiliations:** 1 State Key Laboratory of Transducer Technology, Aerospace Information Research Institute, Chinese Academy of Sciences, Beijing, 100190 People’s Republic of China; 2 School of Future Technology, University of Chinese Academy of Sciences, Beijing, 100049 People’s Republic of China; 3 CAS Key Laboratory of Genomic and Precision Medicine, Collaborative Innovation Center of Genetics and Development, Beijing Institute of Genomics, Chinese Academy of Sciences, Beijing, 100101 People’s Republic of China; 4 China National Center for Bioinformation, Beijing, 100101 People’s Republic of China; 5 School of Electronic, Electrical and Communication Engineering, University of Chinese Academy of Sciences, Beijing, 100049 People’s Republic of China

**Keywords:** Microfluidics, Biosensors

## Abstract

Microfluidic impedance flow cytometry has been widely used in leukocyte differential and counting, but it faces a bottleneck due to the trade-off between impedance detection throughput and sensitivity. In this study, a microfluidic impedance flow cytometer based on a virtual constriction microchannel was reported, in which the virtual constriction microchannel was constructed by crossflow of conductive sample and insulated sheath fluids with underneath micro-electrodes for impedance measurements. Compared to conventional mechanical constriction microchannels, this virtual counterpart could effectively avoid direct physical contact between cells and the microchannel walls to maintain high throughputs, and significantly reduce the volume of the impedance detection region for sensitivity improvements. Using the developed microfluidic impedance flow cytometer, impedance pulses of three leukemia cell lines, K562, Jurkat, and HL-60, were detected, achieving a 99.8% differentiation accuracy through the use of a recurrent neural network. Furthermore, impedance pulses of four white blood cell subpopulations (neutrophils, eosinophils, monocytes, and lymphocytes) from three donors were detected, achieving a classification accuracy of ≥99.2%. A classification network model was established based on purified white blood cell and applied to impedance pulses of two white blood cell mixtures, resulting in proportional distributions of four leukocyte subpopulations within theoretical ranges. These results indicated that the developed microfluidic impedance flow cytometer based on the virtual constriction microchannel could achieve both high detection throughput and high sensitivity, showing great potentials for clinical diagnostics and blood analysis.

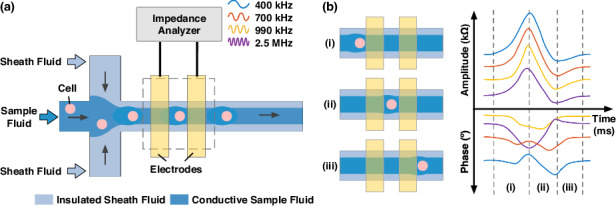

## Introduction

Leukocyte differential and counting has functioned as the first indicator in body status evaluation and clinical examinations of human beings^1,2^. The golden approach of leukocyte differential is the microscopic examination of stained blood smears, which, however, suffers from the key issue of labor intensive and low throughputs^3–9^.

In order to address this issue, flow cytometry has been developed to realize leukocyte differential and counting in an automatic and high-throughput manner^10,11^. Due to the issue of sample losses in cell staining, fluorescent flow cytometry with the staining of a group of antibodies can only be used for leukocyte differential rather than counting^12–16^.

Thus, single-cell electrical and/or optical flow cytometry which is termed as “hematology analyzer” has functioned as the high-throughput approach in this scenario where individual cells with minimal steps of cell treatment travel through a detection cuvette rapidly where electrical impedance and optical scattering are captured for leukocyte differential and counting^11,17–21^. Since the classification of leukocytes is mainly based on single-cell biophysical properties such as membrane capacitance in impedance and nuclear structures in scattering, quality improvements in capturing single-cell impedance and scattering data has been regarded as the driving forces for the developments of hematology analyzers^20,22–24^.

From the perspective of single-cell impedance flow cytometry, it was firstly introduced by Beckman Coulter in the 1950s and realized 3-part leukocyte differential by applying both direct and alternating currents to measure impedance changes caused by cells passing through an aperture^21,25–27^. However, due to the relatively large geometries of detection apertures, conventional Coulter counters suffered from the issue of low detection sensitivities and thus they cannot be directly used to realize 5-part leukocyte differential.

With the developments of microfabrication, detection cuvettes in the dimensions of tens of micrometers can be accurately constructed, leading to improved detection sensitivities of single-cell electrical properties^28–33^. Furthermore, by finely regulating geometrical dimensions and positions of microelectrodes for impedance sampling, high-consistency impedance properties at the single-cell level can be captured regardless of relative positions of single cells within the detection cuvette^34–43^. Leveraging these improvements, microfluidic impedance flow cytometry has been used in blood analysis^44^, tumor^45^, stem cells^46^, alga^47^ and pollen^48^, to name a few. However, still due to relatively low detection sensitivities, these microfluidic impedance flow cytometry has seldomly been used in leukocyte differential^49^.

Aimed to further improve detection sensitivities at the single-cell level, constriction microchannels with cross-sectional areas smaller than single cells were incorporated into microfluidic impedance flow cytometry. In constriction microchannels, single cells are forced to deform through constricted areas and effectively blocked electric lines, generating large impedance variations and improvements in detection sensitivities^50,51^. With the contribution of constriction microchannels, 5-part leukocyte differential was firstly realized based on single-cell impedance flow cytometry only^52^. However, these mechanical constriction microchannels were prone to channel blockage and thus cannot be used commercially for leukocyte differential.

In this study, a virtual constriction microchannel was formed by crossflow of conductive sample and insulated sheath fluids. Different from mechanical constriction microchannels where electric lines and cell traveling were both constricted by solid interfaces, in this study, electric lines were confined by the liquid interface of sample and insulating fluids, which was moveable and thus didn’t restrict the smooth traveling of individual cells. Note that microfluidic impedance flow based on sheath flow focusing were previously used to avoid the coefficient of variation and improve impedance detection sensitivities with a few attempts in Escherichia coli^53^, tumor cells^54,55^ and lymphocytes^56^. Different from these approaches, here the width of the virtual constriction microchannels was comparable with individual cells and thus the interactions between traveling cells and walls of virtual constriction microchannels were carefully studied. In addition, the virtual constriction microchannel was formed by crossflow of conductive sample and insulated sheath fluids, which focused the traveling cells at the center of the channel while concentrating the electric field lines in the sample flow region, producing lower coefficients of variation and higher detection sensitivity. The corresponding impedance profiles due to cell-wall interactions were processed by a deep recurrent neural network to realize the classification of leukocyte subtypes of neutrophils, eosinophils, monocytes and lymphocytes.

## Materials and methodology

### Working principle

**Fig. 1:**
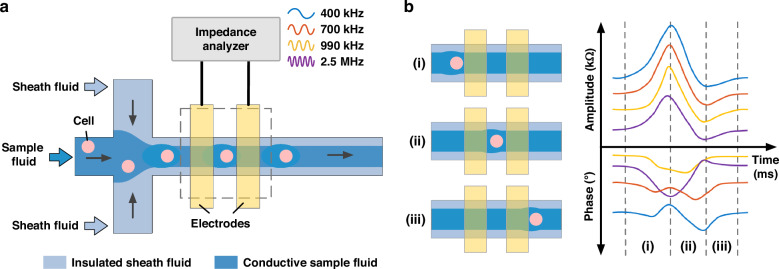
The microfluidic impedance flow cytometer leveraging a virtual constriction microchannel was formed. Working principle of the microfluidic impedance flow cytometer leveraging a virtual constriction microchannel formed by crossflow of conductive sample and insulated sheath fluids with underneath micro-electrodes for impedance measurements, including (**a**) schematic of the microfluidic impedance flow cytometer utilizing virtual constriction microchannel and (**b**) a typcial single-cell pluse and and its formation: as a cell travels through the virtual constriction microchannel between two electrodes, in amplitude, there is a peak due to blockage of electrical lines and then a dip because of expansion of the sample-sheath boundaries due to the traveling cell. As to phase variations, at high frequency domain (e.g., 2.5 MHz), there is a clear dip while at low frequency domain (e.g., 400 kHz), the phase profiles may be affected by the electrical double layer

Figure 1 illustrates the working principle of the microfluidic impedance flow cytometry leveraging the virtual constriction microchannel formed by crossflow of conductive sample and insulated sheath fluids with underneath micro-electrodes for impedance measurements. Compared to mechanical constriction microchannels where electric lines and cell traveling were both constricted by solid interfaces, in this study, electric lines were confined by the liquid interface of sample and insulating fluids, which was moveable and thus didn’t restrict the smooth traveling of individual cells.

The process of single-cell passing through the virtual constriction microchannel could be divided into three parts (see Fig. 1(b)): (i) As the cell first entered the virtual constriction microchannel, the proportion of electric field lines blocked by the cell gradually increased, resulting in a rise in impedance amplitude and a dip in phase at 2.5 MHz. When the cell was fully within the virtual constriction microchannel, changes in both impedance amplitude and phase reached their maximum. (ii) As the cell gradually left the virtual constriction microchannel, the impedance amplitude decreased, corresponding to an increase in impedance phase at 2.5 MHz. When the cell completely left the virtual constriction microchannel, the expansion of the sample-sheath boundaries due to the traveling cell produced minimal values in impedance amplitude and maximal values in impedance phase at 2.5 MHz. (iii) Once the disturbance caused by the cell recovered, impedance amplitude and phase returned to their initial states. Note that at high frequency domain (e.g., 2.5 MHz), there was a clear dip in phase for a traveling cell within the virtual constriction microchannel while at low frequency domain (e.g., 400 kHz), the phase profiles of a traveling cell may be affected by the electrical double layer of coplanar electrodes.

### Materials and cell preparation

The sample fluid was conductive 1x PBS (ThermoFisher), while the sheath fluid was an insulating 10% sucrose solution with osmotic pressure equal to that of the cells. This choice of solutions ensured that the electric field lines were confined within the sample fluid while they had negligible effects on viabilities of individual cells.

Leukemia cell lines of K562, Jurkat, and HL-60 were all purchased from the National Infrastructure of Cell Line Resource. They were then cultured with RPMI-1640 supplemented with 10% fetal bovine serum at a cell incubator (Forma 3111, Thermo Scientific, USA) under 37 °C in 5% CO_2_.

Leukocytes were derived from peripheral bloods of three healthy donors who all signed informed consent forms. After lysing red blood cells, white blood cells were purified by using fluorescent antibody staining combined with flow cytometry (Beckman Coulter). The sorted white blood cell subpopulations were stained with Wright-Giemsa stain (Baso Co.) to confirm high purities of purification (see Supplementary Fig. 1). The leukocyte mixtures were prepared by lysing red blood cells and then kept on standby where Wright-Giemsa staining was conducted for quality control (see Supplementary Fig. 2).

As to device fabrication, the microfluidic device was fabricated using standard processes of soft lithography. The layer of the constriction microchannel was made of PDMS which was molded from photolithography of SU-8 while the electrode layer was fabricated by depositing Cr/Au onto glass slides with photolithography and metal etching. After the plasma treatment, the layer of the constriction microchannel was bonded to the electrode layer to form the microfluidic device.

### Numerical simulation

As to numerical simulation, a 3D simulation model of the virtual constriction microchannel was established using COMSOL Multiphysics 5.5. The analysis incorporated a laminar flow physics, a transport of diluted species physics, and electric current physics. In the laminar flow physics, the inlets of sample and sheath fluids, and the outlet were defined properly, with the channel walls set as “no-slip” boundary conditions and the outlet set at 0 Pa boundary condition. In the transport of diluted species physics, the concentration of the sample fluid was set at 160 mM, and the concentration of the sheath fluid was set at 0 mM. In the electric current physics, the conductivity of the sample fluid was 1.6 S/m, and the conductivity of the sheath fluid was measured at 2.2 × 10^–4^ S/m, with a relative dielectric constant of 78 for both fluids.

Based on grid independence, the width of the generated virtual constriction microchannel was characterized using the concentration distribution and the current density distribution across the cross-section of the impedance detection region. With the sample fluid flow rate fixed at 3 µL/min, the simulation results were compared for sample-to-sheath flow rate ratios of 1/0, 1/0.25, 1/0.5, and 1/1.

### Platform operation

The detection channel had dimensions of 50 µm (width) x 20 µm (height), with an electrode width of 30 µm and an electrode gap of 30 µm, enabling the smooth detection of the majority of leukocytes in healthy peripheral blood samples. In order to obtain consistent results, the electrodes were set 75 µm away from the intersection point of sample and sheath flows where the focused interface was stable and diffusion effects between sample and sheath flows were insignificant. The cells were resuspended in the sample fluid, and the cell concentration of leukemia cell lines was ~5 × 10^5^ cell/mL, while the concentrations for purified leukocyte subpopulations and leukocyte mixtures were 2 ~ 3 × 10^5^ cell/mL.

As to impedance frequencies, the electrical double layer formed at the interface between the electrode surface and the solution dominated at low frequencies, while the parasitic capacitance of impedance measurements dominated at high frequencies. Therefore, to explore the bioelectrical properties of cells, which were composed of cell membrane capacitance and cytoplasmic resistance, four frequencies were selected as 400 kHz, 700 kHz, 990 kHz, and 2.5 MHz. These frequencies, with an effective voltage value of 500 mV, were chosen based on the detection capabilities of the lock-in amplifier (MFLI 5 M, Zurich Instruments) which was used to record impedance pulses caused by cells passing through the virtual constriction microchannel.

### Data analysis

As to data analysis, a recurrent neural network (RNN) was constructed using Matlab R2021b to differentiate impedance pulses of leukocytes. The RNN comprised an input layer, an LSTM layer, a dropout layer, a fully connected layer, a softmax layer, and an output layer. Specifically, the input layer received the amplitude and phase of single-cell impedance pulses at four frequencies, with each cell’s impedance data consisting of 300 time points to prevent the gradient vanishing problem caused by overly long sequences and maintain information richness in the impedance pulses when the sequences were too short.

The LSTM layer, being the key structural layer, contained 64 neurons, an initial learning rate of 0.001, and a batch size dependent on the training size, with the training split into 20 iterations per epoch. The dropout layer, a regularization technique to prevent overfitting, had a dropout rate of 50%. The fully connected layer was connected to the softmax layer to calculate the probabilities of each cell type. The output layer represented the target cell populations, with three types of leukemia cell lines or four types of purified leukocyte subpopulations.

For dataset division, all the data were randomly split into 70% training, 15% validation, and 15% testing. Multiple training sessions were conducted, and the mean differentiation accuracy was taken as the final result and included in the dark gray square at the lower right corner of the confusion matrix. In addition, green numbers showing 100% indicated complete differentiation of the target cells, while green numbers showing 33.3% (for three types) or 25.0% (for four types) indicated a complete inability to differentiate the target cells.

## Results and discussion

### Parameter optimization

**Fig. 2:**
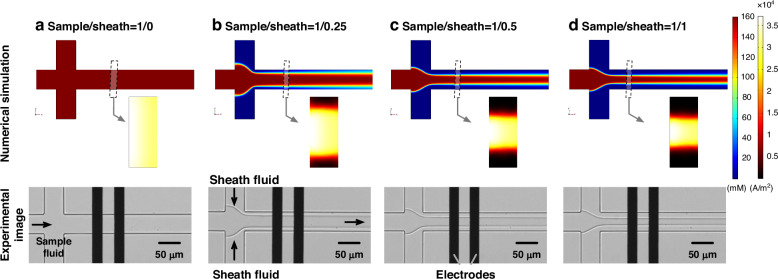
Numerical simulation and experimental images of the constructed virtual constriction microchannels were demonstrated. Numerical simulation and experimental images of the constructed virtual constriction microchannels under sample/sheath ratios of (**a**) 1/0, (**b**) 1/0.25, (**c**) 1/0.5 and (**d**) 1/1. More specifically, in numerical simulation, both concentration and current density distributions were included to evaluate the effects of sample/sheath ratios on the virtual constriction microchannels

Figure 2 presented simulation results and experimental images of the constructed virtual constriction microchannels under sample/sheath ratios of (a) 1/0, (b) 1/0.25, (c) 1/0.5 and (d) 1/1. The simulation results included the distributions of ion concentrations and current densities in the impedance detection region, while the experimental images characterized the width of the virtual constriction microchannel.

In the simulation results, when the sample-to-sheath ratio was 1/0, the ion concentration within the channel was uniformly 160 mM, and the current density distribution spanned the entire microchannel with a width of 50 µm. When the sample-to-sheath ratios were 1/0.25, 1/0.5, and 1/1, the simulated focusing widths were ~34.0 µm, 27.2 µm, and 19.9 µm, respectively. This effectively reduced the volume of the impedance detection region, confining the electric field lines within the virtual constriction microchannel.

In the experimental images, when the sample-to-sheath flow rate ratio was 1/0, the sample fluid was not focused, and no focusing interface between the sample and sheath fluids was observed. When the sample-to-sheath ratio was 1/0.25, a focusing interface between the sample and sheath fluids was present, maintaining stabilities across the microchannel width, with a focusing width of 29.6 ± 0.3 µm. When the sample-to-sheath flow rate ratios were 1/0.5 and 1/1, the focusing widths were 24.2 ± 0.2 µm and 17.0 ± 0.2 µm, respectively. The experimental images demonstrated that the sample fluid could be stably confined in the middle of the impedance detection channel, with the focusing trend consistent with simulation results. Differences in focusing width between simulation and experiments might be attributed to internal channel surface roughness and syringe pump flow rate variations.

As to the width of the virtual constriction microchannel, the smaller width of the virtual constriction microchannel increased disturbances to the focusing interface, producing additional interference within impedance measurements. Besides, the time for cells to pass through the impedance detection region decreased due to the decrease of the virtual constriction microchannels, resulting in fewer data points of a single cell pulse under the condition of the same sampling rate. Thus, the width of the virtual constriction microchannel needed to be greater than the diameter of the cells. Conversely, if the width was too large, the sensitivity of impedance detection decreased. Therefore, a sample-to-sheath flow rate ratio of 1/0.5, with a sheath flow rate of 1.5 µL/min, was selected for subsequent impedance detection. At this ratio, the transit time for a single cell was ~1 ms, with a theoretical detection throughput of ~1000 cell/sec.

### **Leukemia cell lines**

**Fig. 3:**
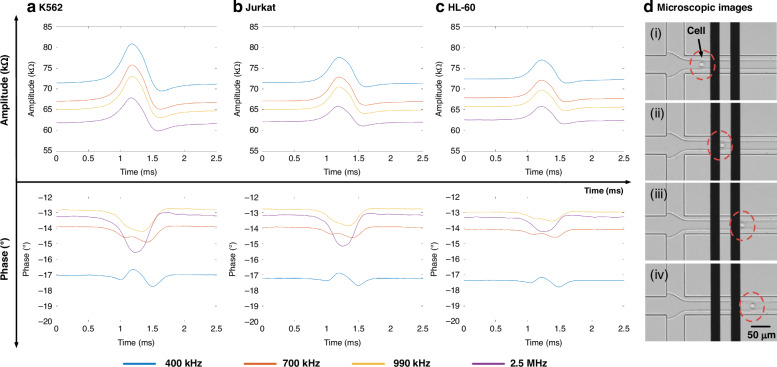
Impedance profiles of three leukemia cell lines were detected and demonstrated. Impedance amplitude and phase profiles of individual (**a**) K562, (**b**) Jurkat, and (**c**) HL-60 traveling through the virtual constriction microchannel with representative microscopic images shown in (**d**) where the expansion of the sample-sheath boundaries due to a traveling cell was noticed

Figure 3 showed the impedance pulses of three leukemia cell lines, including: (a) K562 (a), (b) Jurkat, and (c) HL-60 at four frequencies (400 kHz, 700 kHz, 990 kHz, and 2.5 MHz), along with consecutive microscopic images of a single K562 cell passing through the virtual constriction channel (d).

In terms of impedance amplitude, the impedance pulse of a single cell exhibited an initial rise, followed by a drop, and then a return to the baseline, because a single cell effectively blocked the electric field lines while passing through the virtual constriction microchannel, causing an increase in impedance amplitude. During the cell’s traversal, it disturbed the focusing interface, leading to an expansion of the width of the virtual constriction microchannel. Consequently, when the cell left the impedance detection region, the expanded virtual constriction microchannel caused a drop in impedance amplitude, which then returned to the baseline as the channel width restored (see Fig. 3d).

Regarding impedance phase, the phase waveforms at the four frequencies differed due to the influence of the electrical double layer in the detection circuit, affected by the contact areas between the electrodes and the sample fluid. At 2.5 MHz, the electrical double layer was broken down, resulting in a phase decrease due to the cell membrane capacitance, followed by a slight phase increase due to the expanded local focusing area, and finally returning to the baseline as the focusing width recovered.

Among the impedance pulses, the amplitude ratios at 400 kHz of K562, Jurkat, and HL-60 were 10.79 ± 3.29% (*n*_*cell*_ = 5945), 5.18 ± 2.89% (*n*_*cell*_ = 6457), and 4.41 ± 1.88% (*n*_*cell*_ = 3524) (see Table 1). And the scatter plots of the amplitude ratio distribution at four frequencies were presented in Supplementary Fig. 3. In terms of opacity (2.5 MHz/400 kHz), opacity of K562, Jurkat, and HL-60 were 0.68 ± 0.06, 0.70 ± 0.06, and 0.74 ± 0.06, respectively (see Supplementary Fig. 4). The higher amplitude ratios were obtained with the virtual constriction microchannel despite the enlarged channel dimensions, Compared with that of microfluidic impedance flow cytometry with coplanar electrodes (3%)^57^. The K562 cell line showed the largest proportion of impedance variations, while the HL-60 cell line showed the smallest proportion of impedance variations which gradually decreased with increasing frequency, determined by the dominant roles of the specific capacitance of the cell membrane and the cytoplasmic conductivity. As to the opacity, with few differences among the three cell lines, it is difficult to achieve a highly accurate classification of three cell lines relying on opacity.

**Table 1:** Amplitude ratio, opacity, and cell count of leukemia cell lines, purified WBC subpopulations from three healthy donors at 400 kHz and 2.5 MHz

		Impedance Amplitude Ratio (%)		Opacity (2.5 MHz/400 kHz)	*n*_*cell*_
		400 kHz	2.5 MHz		
K562		10.79 ± 3.29	8.57 ± 2.91	0.68 ± 0.06	5945
Jurkat		5.18 ± 2.89	4.12 ± 2.17	0.70 ± 0.06	6457
HL-60		4.41 ± 1.88	3.72 ± 1.46	0.74 ± 0.06	3524
Donor 1	NEU	2.99 ± 1.40	2.83 ± 1.26	0.81 ± 0.09	685
	EOS	2.46 ± 0.67	2.30 ± 0.66	0.79 ± 0.05	3833
	MON	2.64 ± 1.23	2.40 ± 1.00	0.77 ± 0.05	1535
	LYM	1.30 ± 0.67	1.27 ± 0.71	0.83 ± 0.08	3670
Donor 2	NEU	2.84 ± 1.00	2.63 ± 0.85	0.81 ± 0.05	1537
	EOS	2.71 ± 0.89	2.42 ± 0.77	0.78 ± 0.05	2657
	MON	3.41 ± 2.05	2.96 ± 1.55	0.78 ± 0.06	2853
	LYM	1.35 ± 0.58	1.27 ± 0.55	0.83 ± 0.06	1143
Donor 3	NEU	2.60 ± 1.01	2.42 ± 0.92	0.79 ± 0.04	2218
	EOS	2.34 ± 0.79	2.20 ± 0.73	0.80 ± 0.05	2457
	MON	2.67 ± 1.61	2.46 ± 1.28	0.79 ± 0.05	3735
	LYM	1.11 ± 0.42	1.09 ± 0.40	0.84 ± 0.06	1260

**Fig. 4:**
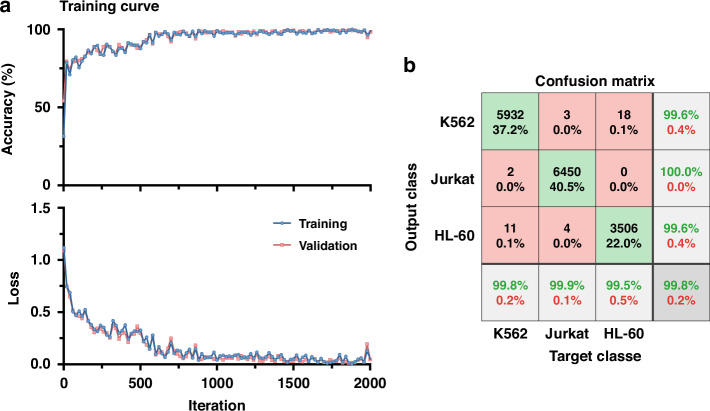
Impedance profiles of three leukemia cell lines were classified. Classification results of leukemia cell lines, including (**a**) classification accuracy and loss versus iteration as well as (**b**) confusion matrix with a 99.8% accuracy in differentiating leukemia cell lines of K562 (*n*_*cell*_ = 5945), Jurkat (*n*_*cell*_ = 6457) and HL-60 (*n*_*cell*_ = 3524)

Figure 4 presented the classification results of K562, Jurkat and HL-60, based on a recurrent neural network, including the training curves composed of classification accuracy *vs*. iteration and loss *vs*. iteration (a) and the confusion matrix (b). The training curves for the training and validation showed no significant differences, indicating no overfitting of the deep neural network. Based on the recurrent neural network, the classification accuracy for K562, Jurkat and HL-60 was 99.8%, with the lowest true positive rate occurring in HL-60 at 99.5% while K562 and Jurkat had the same positive predictive value of 99.6%. The confusion matrices for the training, validation, and testing were shown in supplementary Fig. 5.

### Purified leukocytes

**Fig. 5:**
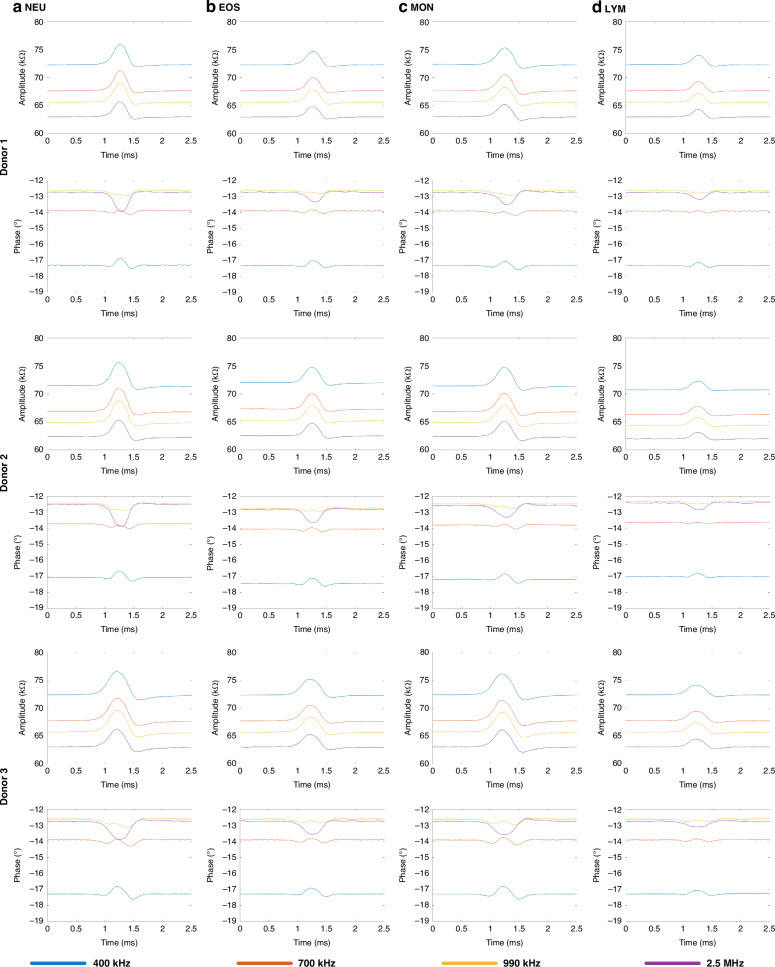
Impedance profiles of four types of leukocytes from three healthy donors were detected and demonstrated. Impedance amplitude and phase profiles of individual (**a**) NEU, (**b**) EOS, (**c**) MON, and (**d**) LYM from Donor 1, Donor 2, and Donor 3 traveling through the virtual constriction microchannel

Figure 5 showed the impedance pulses of four types of leukocytes, including: (a) NEU (a), (b) EOS, (c) MON, and (d) LYM from three healthy donors at four frequencies (400 kHz, 700 kHz, 990 kHz, and 2.5 MHz). In all donors, a leukocyte passing through the virtual constriction microchannel caused an increase in impedance amplitude and a decrease in phase at 2.5 MHz. Due to its smallest cell diameter, LYM exhibited the lowest impedance amplitude.

For donor 1, the impedance amplitude ratios at 400 kHz for NEU, EOS, MON, and LYM were 2.99 ± 1.40% (*n*_*cell*_ = 685), 2.46 ± 0.67% (*n*_*cell*_ = 3833), 2.64 ± 1.23% (*n*_*cell*_ = 1535), and 1.30 ± 0.67% (*n*_*cell*_ = 3670), respectively (see Table 1). The impedance amplitude ratios for LYM at 400 kHz were 1.35 ± 0.58% (*n*_*cell*_ = 1143) and 1.11 ± 0.42% (*n*_*cell*_ = 1260) for donor 2 and donor 3, respectively. The same type of leukocytes exhibited similar impedance amplitude ratios across the three donors, with minor differences possibly attributable to device fabrication errors and individual variations. The scatter plots of the impedance amplitude ratio distributions at four frequencies were presented in Supplementary Fig. 6.

In terms of opacity, the opacities of MON for donor 1, donor 2, and donor 3 were 0.77 ± 0.05 (*n*_*cell*_ = 1535), 0.78 ± 0.06 (*n*_*cell*_ = 2853), and 0.79 ± 0.05 (*n*_*cell*_ = 3735), respectively. The opacities of LYM were 0.83 ± 0.08, 0.83 ± 0.06, and 0.84 ± 0.06 for donor 1, donor 2, and donor 3, respectively. The amplitude ratio and opacity of LYM showed significant differences compared to NEU, EOS, and MON, while the differences among NEU, EOS, and LYM were relatively small. The amplitude ratios and opacities of the leukocyte subpopulations at 400 kHz and 2.5 MHz for the three donors were summarized in Table 1, with the scatter plots of opacity and 400 kHz amplitude ratio distribution shown in Supplementary Fig. 7.

**Fig. 6:**
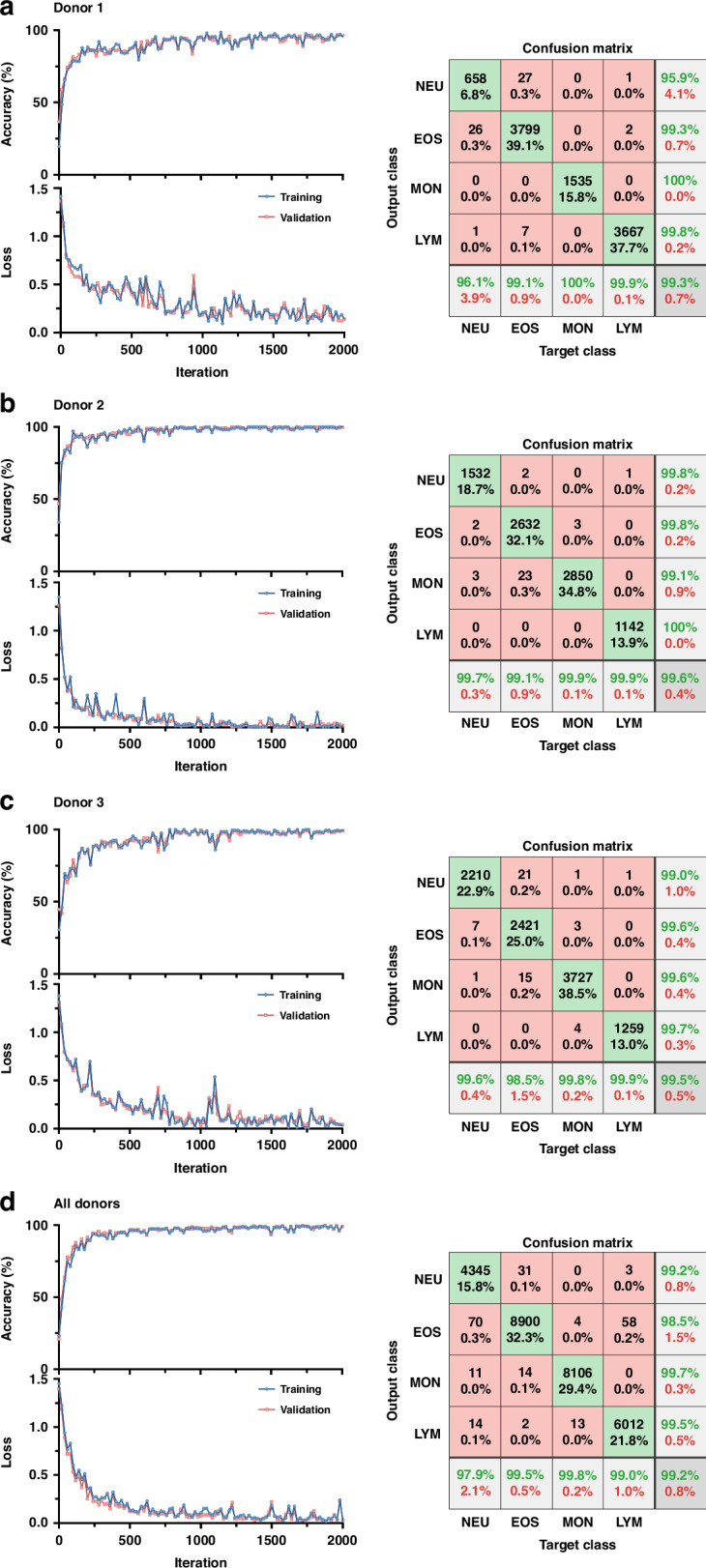
Impedance profiles of four types of leukocytes were classified. Classification accuracy and loss versus iteration as well as confusion matrix in 4-part leukocyte differential of NEU, EOS, MON, and LYM of donor 1 (**a**), donor 2 (**b**), donor 3 (**c**) and all donors (**d**)

Figure 6 presented the classification results of 4-part leukocytes based on a recurrent neural network, including the training curves composed of classification accuracy *vs*. iteration and loss *vs*. iteration, as well as the confusion matrices for (a) donor 1, (b) donor 2, (c) donor 3, and (d) all donors. The training curves for the training and validation showed no significant differences, indicating no overfitting. The classification accuracies for donor 1, donor 2, donor 3, and all donors were 99.3%, 99.6%, 99.5%, and 99.2%, respectively. The lowest true positive rate was observed between NEU and EOS, while the lowest positive predictive value was found among NEU, EOS, and MON. The confusion matrices for the training, validation, and testing were shown in Supplementary Fig. 8.

### Leukocyte mixture

**Fig. 7:**
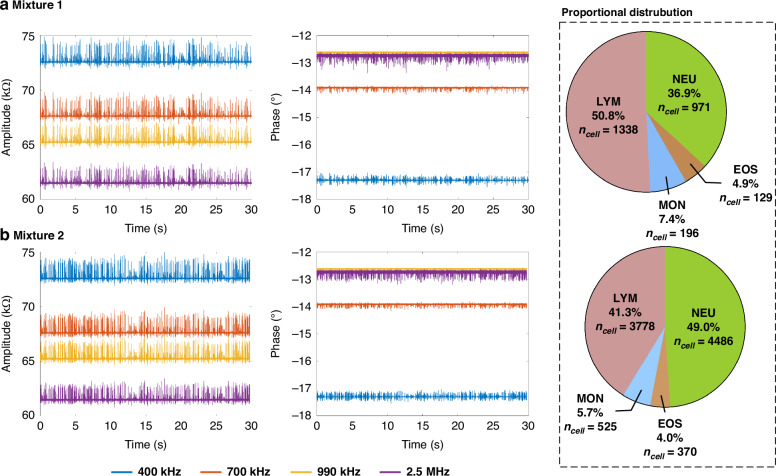
Impedance profiles of two leukocyte mixtures were classified. Impedance amplitude and phase profiles of leukocyte mixture 1 (**a**) and mixture 2 (**b**) with proportional distributions of 4 leukocyte subtypes of NEU, EOS, MON and LYM

Figure 7 presented the 30 s impedance pulses at four frequencies and proportional distribution for mixture 1 (a) and mixture 2 (b). Specifically, impedance pulses were selected when leukocytes passed uniformly through the virtual constriction microchannel to avoid incorrect sorting proportions due to different sedimentation rates of leukocyte subpopulations. No significant specific screening was observed in the impedance pulses of the two leukocyte mixtures, as shown by the impedance magnitude change ratios at 400 kHz in Supplementary Fig. 9.

For the classification of leukocyte mixtures, a classification network model of the recurrent neural network was established based on the impedance pulses of purified leukocyte subpopulations from three donors. This model was then applied to the impedance pulses of the two leukocyte mixtures. In the classification results of the two mixtures, the 4-part WBC fell within the theoretical range and equivalent to the result of blood routine examination, indicating the potential application of the developed microfluidic impedance flow cytometry based on the virtual constriction microchannel in clinical testing.

## Conclusions

This study reported a microfluidic impedance flow cytometer based on the virtual constriction microchannel. Utilizing conductive 1xPBS as the sample fluid and 10% sucrose insulating solution as the sheath fluid, the virtual constriction microchannel was successfully constructed based on sheath focusing. The impedance pulses of three leukemia cell lines, K562, Jurkat, and HL-60, were detected based on this flow cytometer, achieving a classification accuracy of 99.8% using the recurrent neural network. Additionally, the impedance pulses of four types of leukocytes (e.g., NEU, EOS, MON, and LYM), were detected from three healthy donors, achieving a classification accuracy of ≥99.2%. A classification network model was established based on the impedance pulses of purified leukocytes combined with the recurrent neural network and applied to the impedance pulses of two leukocyte mixtures, achieving theoretical range proportions for the 4-part leukocyte differentiation. The microfluidic impedance flow cytometry based on the virtual constriction microchannel provided a viable tool for clinical testing and blood analysis.

## Supplementary information

Supplementary Figures
