# Supplementary material for: Microfluidic impedance flow cytometer leveraging virtual constriction microchannel and its application in leukocyte differential
Source: Microsyst Nanoeng. 2024 Dec 16;10:192. doi: 10.1038/s41378-024-00833-y (PMC11647040; doi:10.1038/s41378-024-00833-y)
Supplement: Supplementary file 1 — Supplementary Figures [file 41378_2024_833_MOESM1_ESM.docx]

**Microfluidic Impedance Flow Cytometer Leveraging Virtual Constriction Microchannel and its Application in Leukocyte Differential**

Minruihong Wang^1,2^*, Jie Zhang^3,4^*, Xiao Chen^1,2^, Yimin Li^1,5^, Xukun Huang^1,5^, Junbo Wang^1,2,5^, Yueying Li^3,4^**, Xiaoye Huo^1,5^**, and Jian Chen^1,2,5^**

^1^State Key Laboratory of Transducer Technology, Aerospace Information Research Institute, Chinese Academy of Sciences, Beijing 100190, People’s Republic of China.

^2^School of Future Technology, University of Chinese Academy of Sciences, Beijing 100049, People’s Republic of China.

^3^CAS Key Laboratory of Genomic and Precision Medicine, Collaborative Innovation Center of Genetics and Development, Beijing Institute of Genomics, Chinese Academy of Sciences, Beijing 100101, People’s Republic of China.

^4^China National Center for Bioinformation, Beijing 100101, People’s Republic of China.

^5^School of Electronic, Electrical and Communication Engineering, University of Chinese Academy of Sciences, Beijing 100049, People’s Republic of China.

*Theses authors contributed equally to this work.

**Correspondence: Yueying Li (liyy@big.ac.cn), Xiaoye Huo (huoxy@aircas.ac.cn), and Jian Chen (chenjian@mail.ie.ac.cn)

**Abstract**

Microfluidic impedance flow cytometry has been widely used in leukocyte differential and counting, but it faces a bottleneck due to the trade-off between impedance detection throughput and sensitivity. In this study, a microfluidic impedance flow cytometer based on a virtual constriction microchannel was reported, in which the virtual constriction microchannel was constructed by crossflow of conductive sample and insulated sheath fluids with underneath micro-electrodes for impedance measurements. Compared to conventional mechanical constriction microchannels, this virtual counterpart could effectively avoid direct physical contact between cells and the microchannel walls to maintain high throughputs, and significantly reduce the volume of the impedance detection region for sensitivity improvements. Using the developed microfluidic impedance flow cytometer, impedance pulses of three leukemia cell lines, K562, Jurkat, and HL-60, were detected, achieving a 99.8% differentiation accuracy through the use of a recurrent neural network. Furthermore, impedance pulses of four white blood cell subpopulations (neutrophils, eosinophils, monocytes, and lymphocytes) from three donors were detected, achieving a classification accuracy of ≥99.2%. A classification network model was established based on purified white blood cell and applied to impedance pulses of two white blood cell mixtures, resulting in proportional distributions of four leukocyte subpopulations within theoretical ranges. These results indicated that the developed microfluidic impedance flow cytometer based on the virtual constriction microchannel could achieve both high detection throughput and high sensitivity, showing great potentials for clinical diagnostics and blood analysis.

**Key Terms:** Virtual Constriction Microchannel, Single-Cell Analysis, Impedance Flow Cytometry, Leukocyte Differential

**Introduction**

Leukocyte differential and counting has functioned as the first indicator in body status evaluation and clinical examinations of human beings [1,2]. The golden approach of leukocyte differential is the microscopic examination of stained blood smears, which, however, suffers from the key issue of labor intensive and low throughputs [3-9].

In order to address this issue, flow cytometry has been developed to realize leukocyte differential and counting in an automatic and high-throughput manner [10,11]. Due to the issue of sample losses in cell staining, fluorescent flow cytometry with the staining of a group of antibodies can only be used for leukocyte differential rather than counting [12-16].

Thus, single-cell electrical and/or optical flow cytometry which is termed as “hematology analyzer” has functioned as the high-throughput approach in this scenario where individual cells with minimal steps of cell treatment travel through a detection cuvette rapidly where electrical impedance and optical scattering are captured for leukocyte differential and counting [17-22]. Since the classification of leukocytes is mainly based on single-cell biophysical properties such as membrane capacitance in impedance and nuclear structures in scattering, quality improvements in capturing single-cell impedance and scattering data has been regarded as the driving forces for the developments of hematology analyzers [23-26].

From the perspective of single-cell impedance flow cytometry, it was firstly introduced by Beckman Coulter in the 1950s and realized 3-part leukocyte differential by applying both direct and alternating currents to measure impedance changes caused by cells passing through an aperture [27-30]. However, due to the relatively large geometries of detection apertures, conventional Coulter counters suffered from the issue of low detection sensitivities and thus they cannot be directly used to realize 5-part leukocyte differential.

With the developments of microfabrication, detection cuvettes in the dimensions of tens of micrometers can be accurately constructed, leading to improved detection sensitivities of single-cell electrical properties [31-36]. Furthermore, by finely regulating geometrical dimensions and positions of microelectrodes for impedance sampling, high-consistency impedance properties at the single-cell level can be captured regardless of relative positions of single cells within the detection cuvette [37-46]. Leveraging these improvements, microfluidic impedance flow cytometry has been used in blood analysis [47], tumor [48], stem cells [49], alga [50] and pollen [51], to name a few. However, still due to relatively low detection sensitivities, these microfluidic impedance flow cytometry has seldomly been used in leukocyte differential [52].

Aimed to further improve detection sensitivities at the single-cell level, constriction microchannels with cross-sectional areas smaller than single cells were incorporated into microfluidic impedance flow cytometry. In constriction microchannels, single cells are forced to deform through constricted areas and effectively blocked electric lines, generating large impedance variations and improvements in detection sensitivities [53,54]. With the contribution of constriction microchannels, 5-part leukocyte differential was firstly realized based on single-cell impedance flow cytometry only [55]. However, these mechanical constriction microchannels were prone to channel blockage and thus cannot be used commercially for leukocyte differential.

In this study, a virtual constriction microchannel was formed by crossflow of conductive sample and insulated sheath fluids. Different from mechanical constriction microchannels where electric lines and cell travelling were both constricted by solid interfaces, in this study, electric lines were confined by the liquid interface of sample and insulating fluids, which was moveable and thus didn’t restrict the smooth travelling of individual cells. Note that microfluidic impedance flow based on sheath flow focusing were previously used to avoid the coefficient of variation and improve impedance detection sensitivities with a few attempts in Escherichia coli [56], tumor cells [57,58] and lymphocytes [59]. Different from these approaches, here the width of the virtual constriction microchannels was comparable with individual cells and thus the interactions between travelling cells and walls of virtual constriction microchannels were carefully studied. In addition, the virtual constriction microchannel was formed by crossflow of conductive sample and insulated sheath fluids, which focused the travelling cells at the center of the channel while concentrating the electric field lines in the sample flow region, producing lower coefficients of variation and higher detection sensitivity. The corresponding impedance profiles due to cell-wall interactions were processed by a deep recurrent neural network to realize the classification of leukocyte subtypes of neutrophils, eosinophils, monocytes and lymphocytes.

**Materials and Methodology**

**Working principle**

Figure 1 illustrates the working principle of the microfluidic impedance flow cytometry leveraging the virtual constriction microchannel formed by crossflow of conductive sample and insulated sheath fluids with underneath micro-electrodes for impedance measurements. Compared to mechanical constriction microchannels where electric lines and cell travelling were both constricted by solid interfaces, in this study, electric lines were confined by the liquid interface of sample and insulating fluids, which was moveable and thus didn’t restrict the smooth travelling of individual cells.

The process of single-cell passing through the virtual constriction microchannel could be divided into three parts (see Figure 1(b)): (i) As the cell first entered the virtual constriction microchannel, the proportion of electric field lines blocked by the cell gradually increased, resulting in a rise in impedance amplitude and a dip in phase at 2.5 MHz. When the cell was fully within the virtual constriction microchannel, changes in both impedance amplitude and phase reached their maximum. (ii) As the cell gradually left the virtual constriction microchannel, the impedance amplitude decreased, corresponding to an increase in impedance phase at 2.5 MHz. When the cell completely left the virtual constriction microchannel, the expansion of the sample-sheath boundaries due to the travelling cell produced minimal values in impedance amplitude and maximal values in impedance phase at 2.5 MHz. (iii) Once the disturbance caused by the cell recovered, impedance amplitude and phase returned to their initial states. Note that at high frequency domain (e.g., 2.5 MHz), there was a clear dip in phase for a travelling cell within the virtual constriction microchannel while at low frequency domain (e.g., 400 kHz), the phase profiles of a travelling cell may be affected by the electrical double layer of coplanar electrodes.

**Materials and Cell Preparation**

The sample fluid was conductive 1x PBS (ThermoFisher), while the sheath fluid was an insulating 10% sucrose solution with osmotic pressure equal to that of the cells. This choice of solutions ensured that the electric field lines were confined within the sample fluid while they had negligible effects on viabilities of individual cells.

Leukemia cell lines of K562, Jurkat, and HL-60 were all purchased from the National Infrastructure of Cell Line Resource. They were then cultured with RPMI-1640 supplemented with 10% fetal bovine serum at a cell incubator (Forma 3111, Thermo Scientific, USA) under 37°C in 5% CO_2_.

Leukocytes were derived from peripheral bloods of three healthy donors who all signed informed consent forms. After lysing red blood cells, white blood cells were purified by using fluorescent antibody staining combined with flow cytometry (Beckman Coulter). The sorted white blood cell subpopulations were stained with Wright-Giemsa stain (Baso Co.) to confirm high purities of purification (see Supplementary Figure 1). The leukocyte mixtures were prepared by lysing red blood cells and then kept on standby where Wright-Giemsa staining was conducted for quality control (see Supplementary Figure 2).

As to device fabrication, the microfluidic device was fabricated using standard processes of soft lithography. The layer of the constriction microchannel was made of PDMS which was molded from photolithography of SU-8 while the electrode layer was fabricated by depositing Cr/Au onto glass slides with photolithography and metal etching. After the plasma treatment, the layer of the constriction microchannel was bonded to the electrode layer to form the microfluidic device.

**Numerical Simulation**

As to numerical simulation, a 3D simulation model of the virtual constriction microchannel was established using COMSOL Multiphysics 5.5. The analysis incorporated a laminar flow physics, a transport of diluted species physics, and electric current physics. In the laminar flow physics, the inlets of sample and sheath fluids, and the outlet were defined properly, with the channel walls set as “no-slip” boundary conditions and the outlet set at 0 Pa boundary condition. In the transport of diluted species physics, the concentration of the sample fluid was set at 160 mM, and the concentration of the sheath fluid was set at 0 mM. In the electric current physics, the conductivity of the sample fluid was 1.6 S/m, and the conductivity of the sheath fluid was measured at 2.2×10⁻⁴ S/m, with a relative dielectric constant of 78 for both fluids.

Based on grid independence, the width of the generated virtual constriction microchannel was characterized using the concentration distribution and the current density distribution across the cross-section of the impedance detection region. With the sample fluid flow rate fixed at 3 µL/min, the simulation results were compared for sample-to-sheath flow rate ratios of 1/0, 1/0.25, 1/0.5, and 1/1.

**Platform Operation**

The detection channel had dimensions of 50 µm (width) x 20 µm (height), with an electrode width of 30 µm and an electrode gap of 30 µm, enabling the smooth detection of the majority of leukocytes in healthy peripheral blood samples. In order to obtain consistent results, the electrodes were set 75 µm away from the intersection point of sample and sheath flows where the focused interface was stable and diffusion effects between sample and sheath flows were insignificant. The cells were resuspended in the sample fluid, and the cell concentration of leukemia cell lines was approximately 5×10⁵ cell/mL, while the concentrations for purified leukocyte subpopulations and leukocyte mixtures were 2~3×10⁵ cell/mL.

As to impedance frequencies, the electrical double layer formed at the interface between the electrode surface and the solution dominated at low frequencies, while the parasitic capacitance of impedance measurements dominated at high frequencies. Therefore, to explore the bioelectrical properties of cells, which were composed of cell membrane capacitance and cytoplasmic resistance, four frequencies were selected as 400 kHz, 700 kHz, 990 kHz, and 2.5 MHz. These frequencies, with an effective voltage value of 500 mV, were chosen based on the detection capabilities of the lock-in amplifier (MFLI 5 M, Zurich Instruments) which was used to record impedance pulses caused by cells passing through the virtual constriction microchannel.

**Data Analysis**

As to data analysis, a recurrent neural network (RNN) was constructed using Matlab R2021b to differentiate impedance pulses of leukocytes. The RNN comprised an input layer, an LSTM layer, a dropout layer, a fully connected layer, a softmax layer, and an output layer. Specifically, the input layer received the amplitude and phase of single-cell impedance pulses at four frequencies, with each cell's impedance data consisting of 300 time points to prevent the gradient vanishing problem caused by overly long sequences and maintain information richness in the impedance pulses when the sequences were too short.

The LSTM layer, being the key structural layer, contained 64 neurons, an initial learning rate of 0.001, and a batch size dependent on the training size, with the training split into 20 iterations per epoch. The dropout layer, a regularization technique to prevent overfitting, had a dropout rate of 50%. The fully connected layer was connected to the softmax layer to calculate the probabilities of each cell type. The output layer represented the target cell populations, with three types of leukemia cell lines or four types of purified leukocyte subpopulations.

For dataset division, all the data were randomly split into 70% training, 15% validation, and 15% testing. Multiple training sessions were conducted, and the mean differentiation accuracy was taken as the final result and included in the dark gray square at the lower right corner of the confusion matrix. In addition, green numbers showing 100% indicated complete differentiation of the target cells, while green numbers showing 33.3% (for three types) or 25.0% (for four types) indicated a complete inability to differentiate the target cells.

**Results and Discussion**

**Parameter Optimization**

Figure 2 presented simulation results and experimental images of the constructed virtual constriction microchannels under sample/sheath ratios of (a) 1/0, (b) 1/0.25, (c) 1/0.5 and (d) 1/1. The simulation results included the distributions of ion concentrations and current densities in the impedance detection region, while the experimental images characterized the width of the virtual constriction microchannel.

In the simulation results, when the sample-to-sheath ratio was 1/0, the ion concentration within the channel was uniformly 160 mM, and the current density distribution spanned the entire microchannel with a width of 50 µm. When the sample-to-sheath ratios were 1/0.25, 1/0.5, and 1/1, the simulated focusing widths were approximately 34.0 µm, 27.2 µm, and 19.9 µm, respectively. This effectively reduced the volume of the impedance detection region, confining the electric field lines within the virtual constriction microchannel.

In the experimental images, when the sample-to-sheath flow rate ratio was 1/0, the sample fluid was not focused, and no focusing interface between the sample and sheath fluids was observed. When the sample-to-sheath ratio was 1/0.25, a focusing interface between the sample and sheath fluids was present, maintaining stabilities across the microchannel width, with a focusing width of 29.6±0.3 µm. When the sample-to-sheath flow rate ratios were 1/0.5 and 1/1, the focusing widths were 24.2±0.2 µm and 17.0±0.2 µm, respectively. The experimental images demonstrated that the sample fluid could be stably confined in the middle of the impedance detection channel, with the focusing trend consistent with simulation results. Differences in focusing width between simulation and experiments might be attributed to internal channel surface roughness and syringe pump flow rate variations.

As to the width of the virtual constriction microchannel, the smaller width of the virtual constriction microchannel increased disturbances to the focusing interface, producing additional interference within impedance measurements. Besides, the time for cells to pass through the impedance detection region decreased due to the decrease of the virtual constriction microchannels, resulting in fewer data points of a single cell pulse under the condition of the same sampling rate. Thus, the width of the virtual constriction microchannel needed to be greater than the diameter of the cells. Conversely, if the width was too large, the sensitivity of impedance detection decreased. Therefore, a sample-to-sheath flow rate ratio of 1/0.5, with a sheath flow rate of 1.5 µL/min, was selected for subsequent impedance detection. At this ratio, the transit time for a single cell was approximately 1 ms, with a theoretical detection throughput of ~1000 cell/sec.

**Leukemia Cell Lines**

Figure 3 showed the impedance pulses of three leukemia cell lines, including: (a) K562 (a), (b) Jurkat, and (c) HL-60 at four frequencies (400 kHz, 700 kHz, 990 kHz, and 2.5 MHz), along with consecutive microscopic images of a single K562 cell passing through the virtual constriction channel (d).

In terms of impedance amplitude, the impedance pulse of a single cell exhibited an initial rise, followed by a drop, and then a return to the baseline, because a single cell effectively blocked the electric field lines while passing through the virtual constriction microchannel, causing an increase in impedance amplitude. During the cell’s traversal, it disturbed the focusing interface, leading to an expansion of the width of the virtual constriction microchannel. Consequently, when the cell left the impedance detection region, the expanded virtual constriction microchannel caused a drop in impedance amplitude, which then returned to the baseline as the channel width restored (see Figure 3(d)).

Regarding impedance phase, the phase waveforms at the four frequencies differed due to the influence of the electrical double layer in the detection circuit, affected by the contact areas between the electrodes and the sample fluid. At 2.5 MHz, the electrical double layer was broken down, resulting in a phase decrease due to the cell membrane capacitance, followed by a slight phase increase due to the expanded local focusing area, and finally returning to the baseline as the focusing width recovered.

Among the impedance pulses, the amplitude ratios at 400 kHz of K562, Jurkat, and HL-60 were 10.79±3.29% (*n_cell_* = 5945), 5.18±2.89% (*n_cell_* = 6457), and 4.41±1.88% (*n_cell_* = 3524) (see Table 1). And the scatter plots of the amplitude ratio distribution at four frequencies were presented in Supplementary Figure 3. In terms of opacity (2.5 MHz/400 kHz), opacity of K562, Jurkat, and HL-60 were 0.68 ± 0.06, 0.70 ± 0.06, and 0.74 ± 0.06, respectively (see Supplementary Figure 4). The higher amplitude ratios were obtained with the virtual constriction microchannel despite the enlarged channel dimensions, Compared with that of microfluidic impedance flow cytometry with coplanar electrodes (3%) [60]. The K562 cell line showed the largest proportion of impedance variations, while the HL-60 cell line showed the smallest proportion of impedance variations which gradually decreased with increasing frequency, determined by the dominant roles of the specific capacitance of the cell membrane and the cytoplasmic conductivity. As to the opacity, with few differences among the three cell lines, it is difficult to achieve a highly accurate classification of three cell lines relying on opacity.

Figure 4 presented the classification results of K562, Jurkat and HL-60, based on a recurrent neural network, including the training curves composed of classification accuracy *vs.* iteration and loss *vs.* iteration (a) and the confusion matrix (b). The training curves for the training and validation showed no significant differences, indicating no overfitting of the deep neural network. Based on the recurrent neural network, the classification accuracy for K562, Jurkat and HL-60 was 99.8%, with the lowest true positive rate occurring in HL-60 at 99.5% while K562 and Jurkat had the same positive predictive value of 99.6%. The confusion matrices for the training, validation, and testing were shown in supplementary figure 5.

**Purified Leukocytes**

Figure 5 showed the impedance pulses of four types of leukocytes, including: (a) NEU (a), (b) EOS, (c) MON, and (d) LYM from three healthy donors at four frequencies (400 kHz, 700 kHz, 990 kHz, and 2.5 MHz). In all donors, a leukocyte passing through the virtual constriction microchannel caused an increase in impedance amplitude and a decrease in phase at 2.5 MHz. Due to its smallest cell diameter, LYM exhibited the lowest impedance amplitude.

For donor 1, the impedance amplitude ratios at 400 kHz for NEU, EOS, MON, and LYM were 2.99±1.40% (*n_cell_* = 685), 2.46±0.67% (*n_cell_* = 3833), 2.64±1.23% (*n_cell_* = 1535), and 1.30±0.67% (*n_cell_* = 3670), respectively (see Table 1). The impedance amplitude ratios for LYM at 400 kHz were 1.35±0.58% (*n_cell_* = 1143) and 1.11±0.42% (*n_cell_* = 1260) for donor 2 and donor 3, respectively. The same type of leukocytes exhibited similar impedance amplitude ratios across the three donors, with minor differences possibly attributable to device fabrication errors and individual variations. The scatter plots of the impedance amplitude ratio distributions at four frequencies were presented in Supplementary Figure 6.

In terms of opacity, the opacities of MON for donor 1, donor 2, and donor 3 were 0.77 ±0.05 (*n_cell_*=1535), 0.78±0.06 (*n_cell_*=2853), and 0.79±0.05 (*n_cell_*=3735), respectively. The opacities of LYM were 0.83 ± 0.08, 0.83 ± 0.06, and 0.84 ± 0.06 for donor 1, donor 2, and donor 3, respectively. The amplitude ratio and opacity of LYM showed significant differences compared to NEU, EOS, and MON, while the differences among NEU, EOS, and LYM were relatively small. The amplitude ratios and opacities of the leukocyte subpopulations at 400 kHz and 2.5 MHz for the three donors were summarized in Table 1, with the scatter plots of opacity and 400 kHz amplitude ratio distribution shown in Supplementary Figure 7.

Figure 6 presented the classification results of 4-part leukocytes based on a recurrent neural network, including the training curves composed of classification accuracy *vs.* iteration and loss *vs.* iteration, as well as the confusion matrices for (a) donor 1, (b) donor 2, (c) donor 3, and (d) all donors. The training curves for the training and validation showed no significant differences, indicating no overfitting. The classification accuracies for donor 1, donor 2, donor 3, and all donors were 99.3%, 99.6%, 99.5%, and 99.2%, respectively. The lowest true positive rate was observed between NEU and EOS, while the lowest positive predictive value was found among NEU, EOS, and MON. The confusion matrices for the training, validation, and testing were shown in Supplementary Figure 8.

**Leukocyte Mixture**

Figure 7 presented the 30s impedance pulses at four frequencies and proportional distribution for mixture 1 (a) and mixture 2 (b). Specifically, impedance pulses were selected when leukocytes passed uniformly through the virtual constriction microchannel to avoid incorrect sorting proportions due to different sedimentation rates of leukocyte subpopulations. No significant specific screening was observed in the impedance pulses of the two leukocyte mixtures, as shown by the impedance magnitude change ratios at 400 kHz in Supplementary Figure 9.

For the classification of leukocyte mixtures, a classification network model of the recurrent neural network was established based on the impedance pulses of purified leukocyte subpopulations from three donors. This model was then applied to the impedance pulses of the two leukocyte mixtures. In the classification results of the two mixtures, the 4-part WBC fell within the theoretical range and equivalent to the result of blood routine examination, indicating the potential application of the developed microfluidic impedance flow cytometry based on the virtual constriction microchannel in clinical testing.

**Conclusions**

This study reported a microfluidic impedance flow cytometer based on the virtual constriction microchannel. Utilizing conductive 1xPBS as the sample fluid and 10% sucrose insulating solution as the sheath fluid, the virtual constriction microchannel was successfully constructed based on sheath focusing. The impedance pulses of three leukemia cell lines, K562, Jurkat, and HL-60, were detected based on this flow cytometer, achieving a classification accuracy of 99.8% using the recurrent neural network. Additionally, the impedance pulses of four types of leukocytes (e.g., NEU, EOS, MON, and LYM), were detected from three healthy donors, achieving a classification accuracy of ≥99.2%. A classification network model was established based on the impedance pulses of purified leukocytes combined with the recurrent neural network and applied to the impedance pulses of two leukocyte mixtures, achieving theoretical range proportions for the 4-part leukocyte differentiation. The microfluidic impedance flow cytometry based on the virtual constriction microchannel provided a viable tool for clinical testing and blood analysis.

**Acknowledgement**

This work was supported by the Grants of 62331025 and 62121003 from the National Natural Science Foundation of China.

**Competing interests**

The authors declare no conflicts of interest

**References**

1. Greer J, Arber DA, Glader BE, List AF, Means R, Rodgers GM, et al. Wintrobe's clinical hematology: fourteenth edition. 2018. 1-7072.
2. Vuckovic D, Bao EL, Akbari P, Lareau CA, Mousas A, Jiang T, et al. The polygenic and monogenic basis of blood traits and diseases. Cell 2020; 182: 1214-1231.
3. Mohammed EA, Mohamed MM, Far BH and Naugler C, et al. Peripheral blood smear image analysis: A comprehensive review. J Pathol Inform 2014; 5: 9.
4. Patel N, Mishra A. Automated leukaemia detection using microscopic images. Procedia Computer Science 2015; 58: 635-642.
5. Merino A, Puigvi L, Boldu L, Alferez S, Rodellar J. Optimizing morphology through blood cell image analysis. Int J Lab Hematol 2018; 40: 54-61.
6. Matek C, Schwarz S, Spiekermann K, Marr C. Human-level recognition of blast cells in acute myeloid leukaemia with convolutional neural networks. Nat Mach Intell 2019; 1: 538-544.
7. Sidhom JW, Siddarthan IJ, Lai BS, Luo A, Hambley BC, Bynum J, et al. Deep learning for diagnosis of acute promyelocytic leukemia via recognition of genomically imprinted morphologic features. NPJ Precis Oncol 2021; 5: 38.
8. Das PK, Meher S. An efficient deep convolutional neural network based detection and classification of acute lymphoblastic leukemia. Expert Syst Appl 2021; 183: 115311.
9. Rastogi P, Khanna K, Singh V. LeuFeatx: deep learning-based feature extractor for the diagnosis of acute leukemia from microscopic images of peripheral blood smear. Comput Biol Med 2022; 142: 105236.
10. Shah A, Naqvi SS, Naveed K, Salem N, Khan MAU, Alimgeer KS. Automated diagnosis of leukemia: A comprehensive review. IEEE Access 2021; 9: 132097-132124.
11. Luo J, Chen C, Li Q. White blood cell counting at point-of-care testing: a review. Electrophoresis 2020; 41: 1450-1468.
12. Hübl W, Wolfbauer G, Andert S, et al. Toward a new reference method, for the leukocyte five-part differential. Cytometry 1997; 30: 72-84.
13. Suzuki S, Eguchi N. Leukocyte differential analysis in multiple laboratory species by a laser multi-angle polarized light scattering separation method. Experimental Animals 1999; 48: 107-114.
14. Luc J, Lacronique C, Frebet E, et al. "6 markers/5 colors" extended white blood cell differential by flow cytornetry. Cytometry Part A 2007; 71(11): 934-944.
15. Bjornsson S, Wahlstrom S, Norstrom E, et al. Total nucleated cell differential for blood and bone marrow using a single tube in a five-color flow cytometer. Cytometry Part B 2008; 74(2): 91-103.
16. Takagi Y, Kono M, Yamamoto S, et al. Comparison of optical data from flow cytometry and microscopy of leukocytes after exposure to specific reagents. Microscopy 2015; 64: 305-310.
17. Buttarello M, Plebani M. Automated blood cell counts: State of the art. Am J Clin Pathol 2008; 130: 104-116.
18. DeNicola DB. Advances in hematology analyzers. Top Companion Anim M 2011; 26: 52-61.
19. Green R, Wachsmann-Hogiu S. Development, history, and future of automated cell counters. Clin. Lab. Med. 2015; 35: 1-10.
20. Cembrowski GS, Clarke G. Quality control of automated cell counters. Clin. Lab. Med. 2015; 35: 59-71.
21. Don M. The coulter principle: Foundation of an industry. J Lab Autom 2016; 8: 72-81.
22. Luo J, Chen C, Li Q. White blood cell counting at point-of-care testing: a review. Electrophoresis 2020; 41: 1450-1468.
23. D. S. Chabot-Richards, and T. I. George. White blood cell counts reference methodology, Clinics in Laboratory Medicine 2015; 35(1): 11-24.
24. J. Y. Vis, and A. Huisman, Verification and quality control of routine hematology analyzers, International Journal of Laboratory Hematology 2016; 38: 100-109.
25. G. S. Cembrowski, and G. Clarke. Quality control of automated cell counters. Clinics in Laboratory Medicine 2015; 35(1): 59-71.
26. J. Chen, and Y. Lu. Biosensors for single-cell analysis. Academic Press 2022; DOI: https://doi.org/10.1016/C2020-0-02901-4.
27. Don M. The coulter principle: Foundation of an industry. J Lab Autom 2016; 8: 72-81.
28. Graham MD. The coulter principle: A history. Cytom Part A 2022; 101: 8-11.
29. Carey TR, Cotner KL, Li B, Sohn LL. Developments in label-free microfluidic methods for single-cell analysis and sorting. Wires Nanomed Nanobi 2019; 11: e1529.
30. Zhang Z, Huang X, Liu K, Lan T, Wang Z, Zhu Z, Recent advances in electrical impedance sensing technology for single-cell analysis. Biosensors 2021; 11(11): 470.
31. Honrado C, Bisegna P, Swami NS, Caselli F. Single-cell microfluidic impedance cytometry: From raw signals to cell phenotypes using data analytics. Lab Chip 2021; 21: 22-54.
32. Daguerre H, Solsona M, Cottet J, Gauthier M, Renaud P, Bolopion A. Positional dependence of particles and cells in microfluidic electrical impedance flow cytometry: Origin, challenges and opportunities. Lab Chip 2020; 20: 3665-3689.
33. Wu H, Zhu J, Huang Y, et al. Microfluidic-based single-cell study: current status and future perspective. Molecules 2018; 23: 2347.
34. Yang R, Fu L, Hou H. Review and perspectives on microfluidic flow cytometers. Sensors and Actuators B: Chemical 2018; 266: 26-45.
35. Wang M, Liang H, Chen X, Chen D, Wang J, Zhang Y, et al. Developments of conventional and microfluidic flow cytometry enabling high-throughput characterization of single cells. Biosensors 2022; 12: 443.
36. Z. Zhou, Y. Chen, S. Zhu, et al., " Inertial microfluidics for high-throughput cell analysis and detection: a review," Analyst 2021; 146(20): 6064-6083.
37. S. Zhu, X. Zhang, Z. Zhou, et al., Microfluidic impedance cytometry for single-cell sensing: Review on electrode configurations. Talanta 2021; 233: 122571.
38. Zhang W, Hu Y, Choi G, Liang S, Liu M, Guan W. Microfluidic multiple cross-correlated coulter counter for improved particle size analysis. Sens. Actuators B Chem. 2019; 296: 126615.
39. De Ninno A, Reale R, Giovinazzo A, Bertani FR, Businaro L, Bisegna P, et al. High-throughput label-free characterization of viable, necrotic and apoptotic human lymphoma cells in a coplanar-electrode microfluidic impedance chip. Biosens Bioelectron 2020; 150: 111887.
40. Petchakup C, Yang H, Gong L, et al. Microfluidic impedance-deformability cytometry for label-Free single neutrophil mechanophenotyping. Small 2022; 18(18): e2104822.
41. Spencer D, Morgan H. High speed single cell dielectric spectroscopy. ACS Sens 2020; 5: 423-430.
42. de Bruijn DS, Jorissen KFA, Olthuis W, van den Berg A. Determining particle size and position in a coplanar electrode setup using measured opacity for microfluidic cytometry. Biosensors 2021; 11(10): 353.
43. Zhong J, Liang M and Ai Y. Submicron-precision particle characterization in microfluidic impedance cytometry with double differential electrodes. Lab Chip 2021; 21: 2869-2880.
44. Tang T, Liu X, Kiya R, Shen Y, Yuan Y, Zhang T, et al. Microscopic impedance cytometry for quantifying single cell shape. Biosens Bioelectron 2021; 193: 113521.
45. Caselli F, De Ninno A, Reale R, Businaro L, Bisegna P. A bayesian approach for coincidence resolution in microfluidic impedance cytometry. IEEE. Trans. Biomed. Eng. 2021; 68: 340-349.
46. Zhou C, Shen H, Feng H, et al., Enhancing signals of microfluidic impedance cytometry through optimization of microelectrode array. Electrophoresis 2022; 246: 1-9.
47. Peng T, Su X, Cheng X, Wei Z, Su X, Li Q. A microfluidic cytometer for white blood cell analysis. Cytometry A 2021; 99(11): 1107-1113.
48. Honrado C, Salahi A, Adair SJ, Moore JH, Bauer TW, Swami NS. Automated biophysical classification of apoptotic pancreatic cancer cell subpopulations by using machine learning approaches with impedance cytometry. Lab Chip 2022; 22(19): 3708-3720.
49. Gong L, He L, Lu N, Petchakup C, Li KHH, Tay CY, Hou HW. Label-free single microparticles and cell aggregates sorting in continuous cell-based manufacturing. Adv Healthc Mater 2024: 2304529.
50. Chen X, Shen M, Liu S, Wu C, Sun L, Song Z, Shi J, Yuan Y, Zhao Y. Microfluidic impedance cytometry with flat-end cylindrical electrodes for accurate and fast analysis of marine microalgae. Lab Chip 2024; 24(7): 2058-2068.
51. M. D'Orazio, R. Reale, A. De Ninno, et al., ElectroOptical classification of pollen grains via microfluidics and machine learning. in IEEE Transactions on Biomedical Engineering 2021; 69(2): 921-931.
52. Han X, van Berkel C, Gwyer J, Capretto L, Morgan H. Microfluidic lysis of human blood for leukocyte analysis using single cell impedance cytometry. Anal Chem 2012; 84(2): 1070-1075.
53. Y. Zhang, H. Liang, H. Tan, D. Chen, Y. Wang, Y. Xu, J. Wang, and J. Chen, “Development of microfluidic platform to high-throughput quantify single-cell intrinsic bioelectrical markers of tumor cell lines, subtypes and patient tumor cells,” Sensors and Actuators B: Chemical 2020; 317: 128231.
54. Zhang Y, Wang M, Zheng Y, Chen D, Wang W, Wang J, et al. A microfluidic platform for characterizing single-cell intrinsic bioelectrical properties with large sample size. IEEE Trans. Electron Devices 2022; 69: 5177-5184.
55. Wang M, Tan H, Li Y, Chen X, Chen D, Wang J, et al. Toward five-part differential of leukocytes based on electrical impedances of single cells and neural network. Cyto Part A 2023; 103(5): 439-446.
56. Zhu J, Feng Y, Chai H, et al. Performance-enhanced clogging-free viscous sheath constriction impedance flow cytometry. Lab Chip 2023; 23(11): 2531-2539.
57. Zhou Y, Wang J, Liu T, et al. An adaptive three-dimensional hydrodynamic focusing microfluidic impedance flow cytometer. Analyst 2023; 148: 3239-3246.
58. Ni C, Yang M, Yang S, et al. Three-dimensional inertial focusing based impedance cytometer enabling high-accuracy characterization of electrical properties of tumor cells. Lab Chip. 2024; 24(18):4333-4343.
59. Watkins N, Venkatesan BM, Toner M, et al. A robust electrical microcytometer with 3-dimensional hydrofocusing. Lab Chip. 2009; 9(22): 3177-3184.
60. Gawad S, Schild L, Renaud PH. Micromachined impedance spectroscopy flow cytometer for cell analysis and particle sizing. Lab Chip 2001;1(1):76-82.


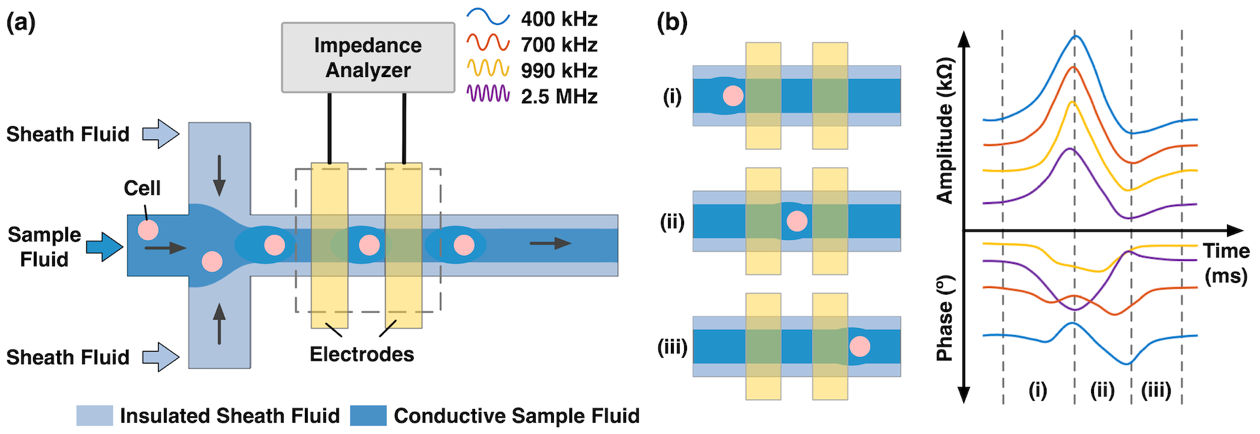


**Figure 1. The microfluidic impedance flow cytometer leveraging a virtual constriction microchannel was formed.** (a) Working principle of the microfluidic impedance flow cytometer leveraging a virtual constriction microchannel formed by crossflow of conductive sample and insulated sheath fluids with underneath micro-electrodes for impedance measurements. (b) As a cell travels through the virtual constriction microchannel between two electrodes, in amplitude, there is a peak due to blockage of electrical lines and then a dip because of expansion of the sample-sheath boundaries due to the travelling cell. As to phase variations, at high frequency domain (e.g., 2.5 MHz), there is a clear dip while at low frequency domain (e.g., 400 kHz), the phase profiles may be affected by the electrical double layer.


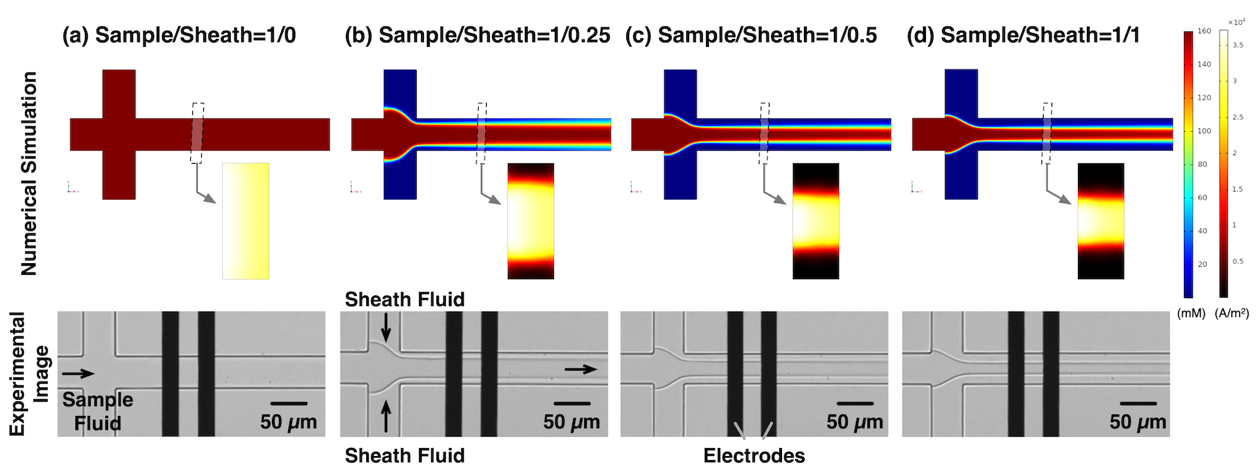


**Figure 2.** **Numerical simulation and experimental images of the constructed virtual constriction microchannels were demonstrated.** Numerical simulation and experimental images with sample/sheath ratios of (a) 1/0, (b) 1/0.25, (c) 1/0.5 and (d) 1/1. More specifically, in numerical simulation, both concentration and current density distributions were included to evaluate the effects of sample/sheath ratios on the virtual constriction microchannels.


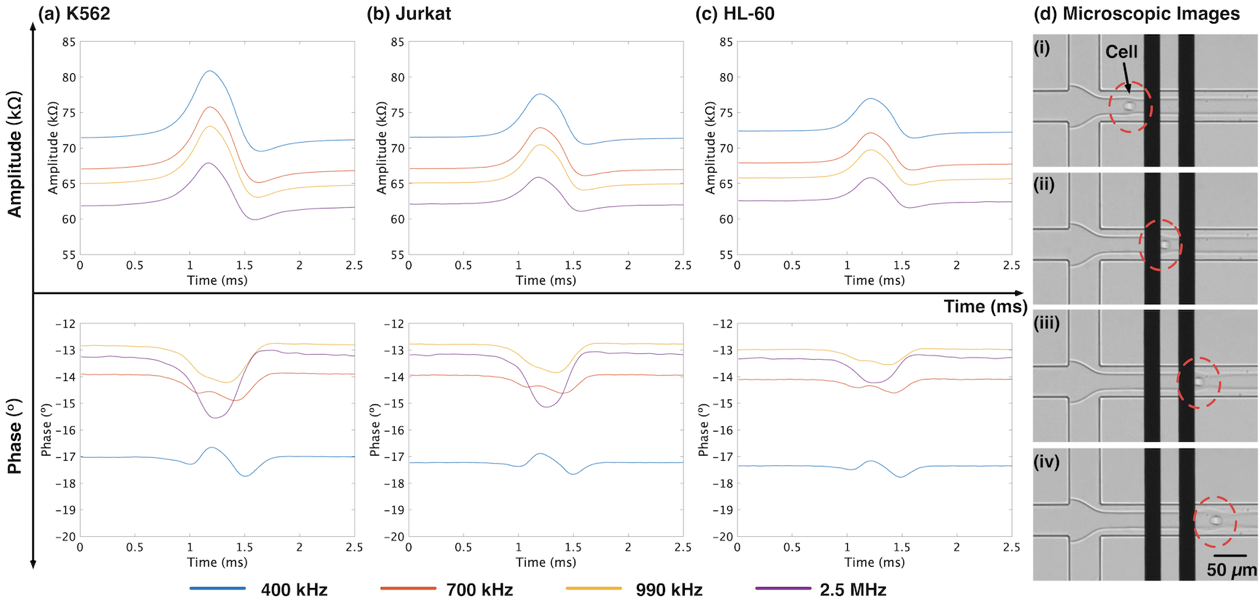


**Figure 3.** **Impedance profiles of three leukemia cell lines were detected and demonstrated.** Impedance amplitude and phase profiles of individual (a) K562, (b) Jurkat, and (c) HL-60 travelling through the virtual constriction microchannel with representative microscopic images shown in (d) where the expansion of the sample-sheath boundaries due to a travelling cell was noticed.


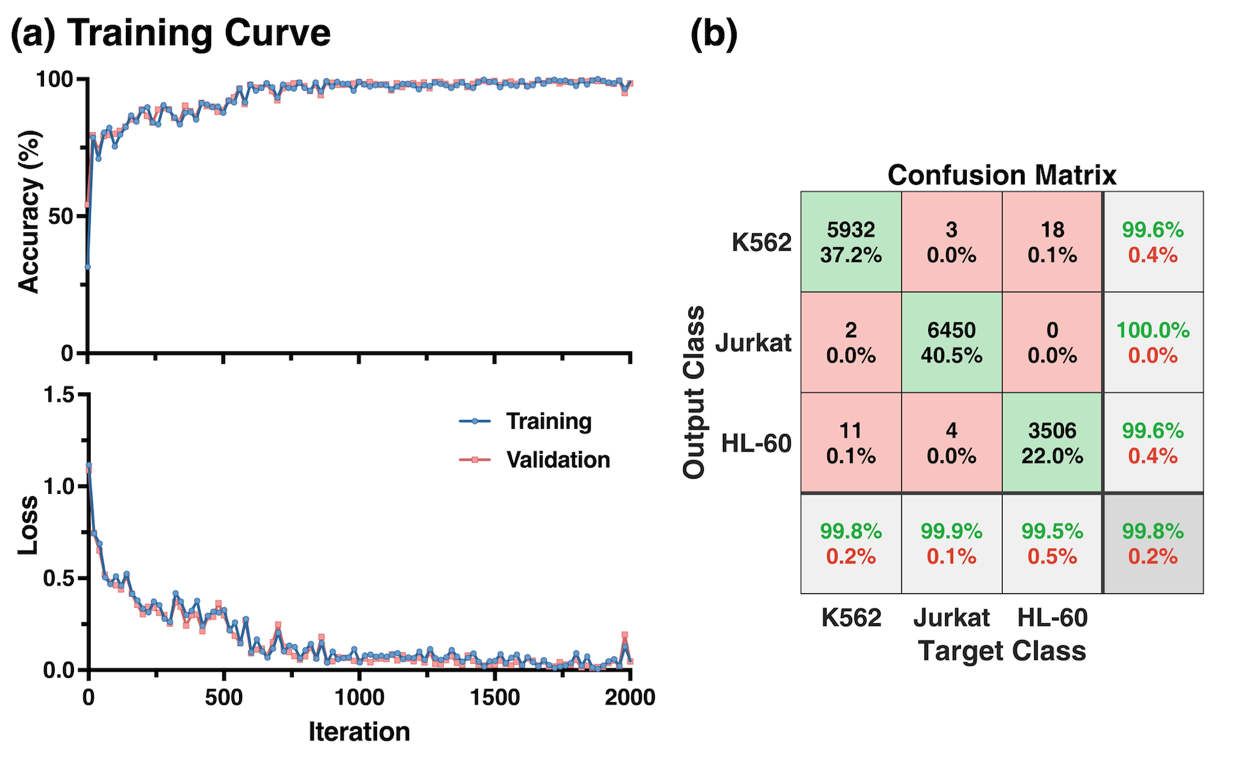


**Figure 4. Impedance profiles of three leukemia cell lines were classified.** (a) Classification accuracy and loss versus iteration as well as (b) confusion matrix with a 99.8% accuracy in differentiating leukemia cell lines of K562 (*n_cell_* = 5945), Jurkat (*n_cell_* = 6457) and HL-60 (*n_cell_* = 3524).


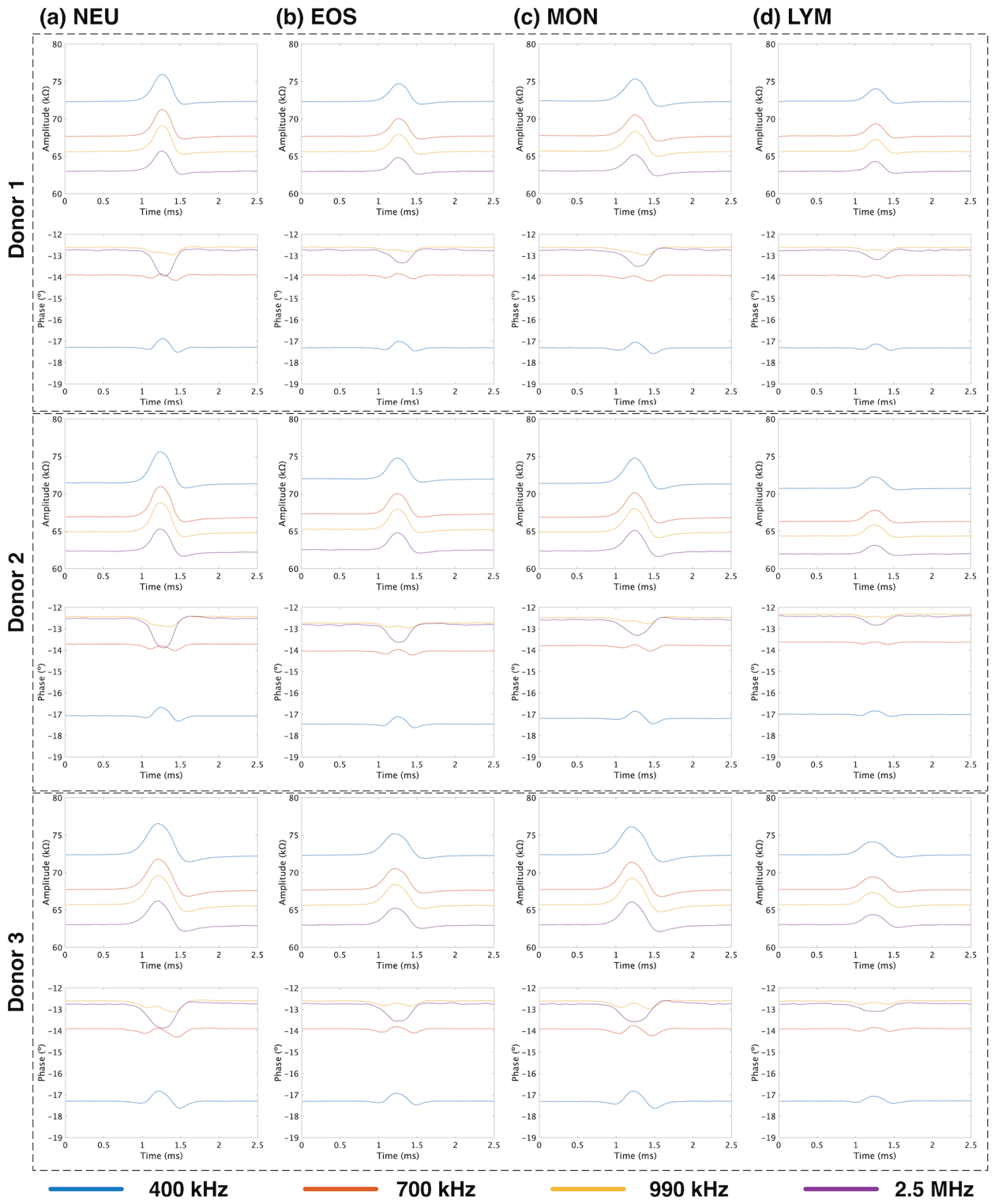


**Figure 5. Impedance profiles of four types of leukocytes from three healthy donors were detected and demonstrated.** Impedance amplitude and phase profiles of individual (a) NEU, (b) EOS, (c) MON, and (d) LYM from Donor 1, Donor 2, and Donor 3 travelling through the virtual constriction microchannel.


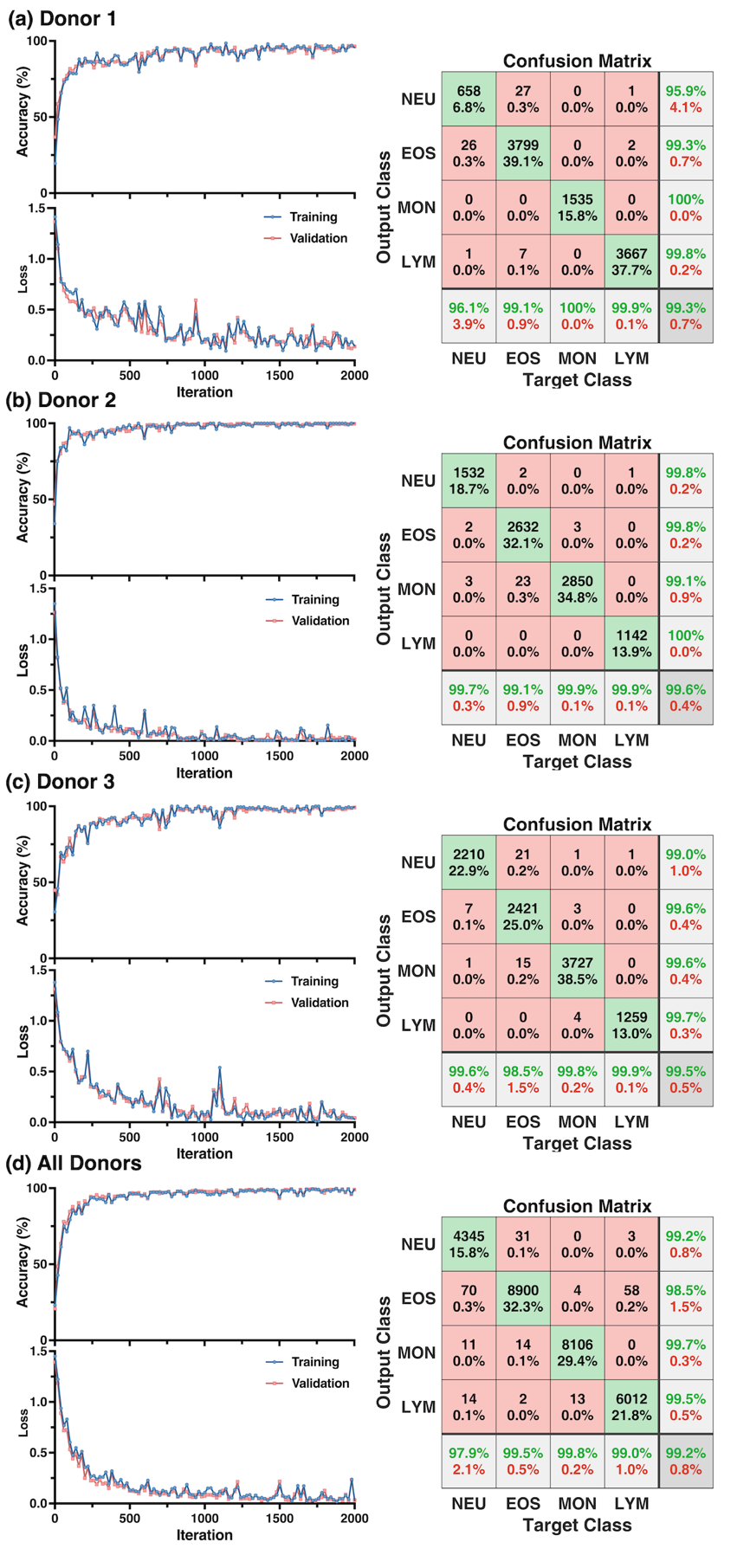


**Figure 6. Impedance profiles of four types of leukocytes were classified.** Classification accuracy and loss versus iteration as well as confusion matrix in 4-part leukocyte differential of NEU, EOS, MON, and LYM of donor 1 (a), donor 2 (b), donor 3 (c) and all donors (d).


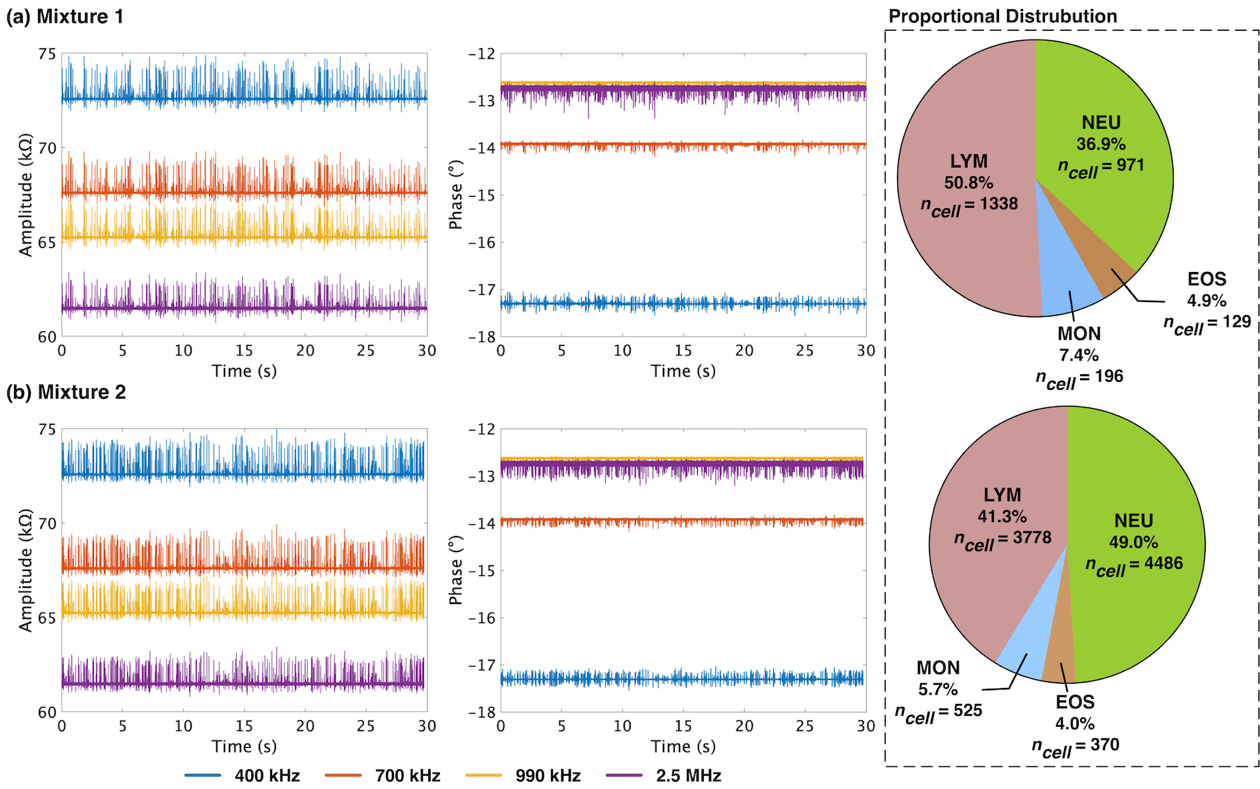


**Figure 7. Impedance profiles of two leukocyte mixtures were classified.** Impedance amplitude and phase profiles of leukocyte mixture 1 (a) and mixture 2 (b) with proportional distributions of 4 leukocyte subtypes of NEU, EOS, MON and LYM.

**Table 1.** Amplitude ratio, opacity, and cell count of leukemia cell lines, purified WBC subpopulations from three healthy donors at 400 kHz and 2.5 MHz.

|  | | Impedance Amplitude Ratio (%) | | Opacity  (2.5MHz/400kHz) | *n_cell_* |
| --- | --- | --- | --- | --- | --- |
|  |  | 400 kHz | 2.5 MHz |  |  |
| K562 | | 10.79±3.29 | 8.57±2.91 | 0.68±0.06 | 5945 |
| Jurkat | | 5.18±2.89 | 4.12±2.17 | 0.70±0.06 | 6457 |
| HL-60 | | 4.41±1.88 | 3.72±1.46 | 0.74±0.06 | 3524 |
| Donor 1 | NEU | 2.99±1.40 | 2.83±1.26 | 0.81±0.09 | 685 |
|  | EOS | 2.46±0.67 | 2.30±0.66 | 0.79±0.05 | 3833 |
|  | MON | 2.64±1.23 | 2.40±1.00 | 0.77±0.05 | 1535 |
|  | LYM | 1.30±0.67 | 1.27±0.71 | 0.83±0.08 | 3670 |
| Donor 2 | NEU | 2.84±1.00 | 2.63±0.85 | 0.81±0.05 | 1537 |
|  | EOS | 2.71±0.89 | 2.42±0.77 | 0.78±0.05 | 2657 |
|  | MON | 3.41±2.05 | 2.96±1.55 | 0.78±0.06 | 2853 |
|  | LYM | 1.35±0.58 | 1.27±0.55 | 0.83±0.06 | 1143 |
| Donor 3 | NEU | 2.60±1.01 | 2.42±0.92 | 0.79±0.04 | 2218 |
|  | EOS | 2.34±0.79 | 2.20±0.73 | 0.80±0.05 | 2457 |
|  | MON | 2.67±1.61 | 2.46±1.28 | 0.79±0.05 | 3735 |
|  | LYM | 1.11±0.42 | 1.09±0.40 | 0.84±0.06 | 1260 |


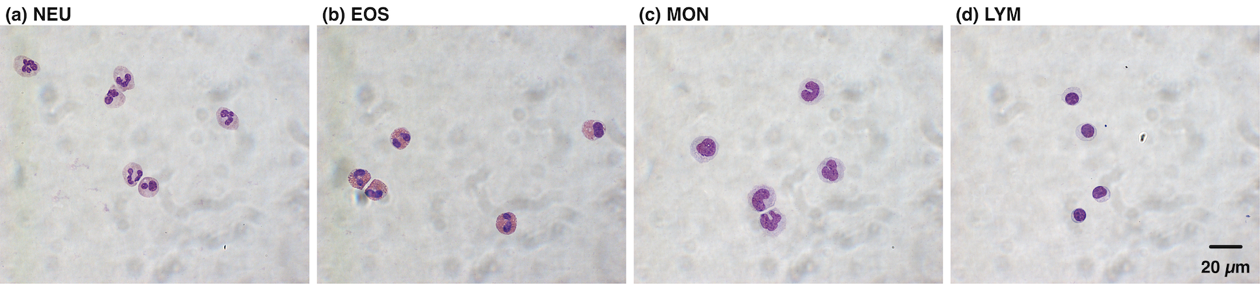


**Supplementary Figure 1.** Stained microscopic images of (a) NEU, (b) EOS, (c) MON, and (d) LYM after leukocyte purification.


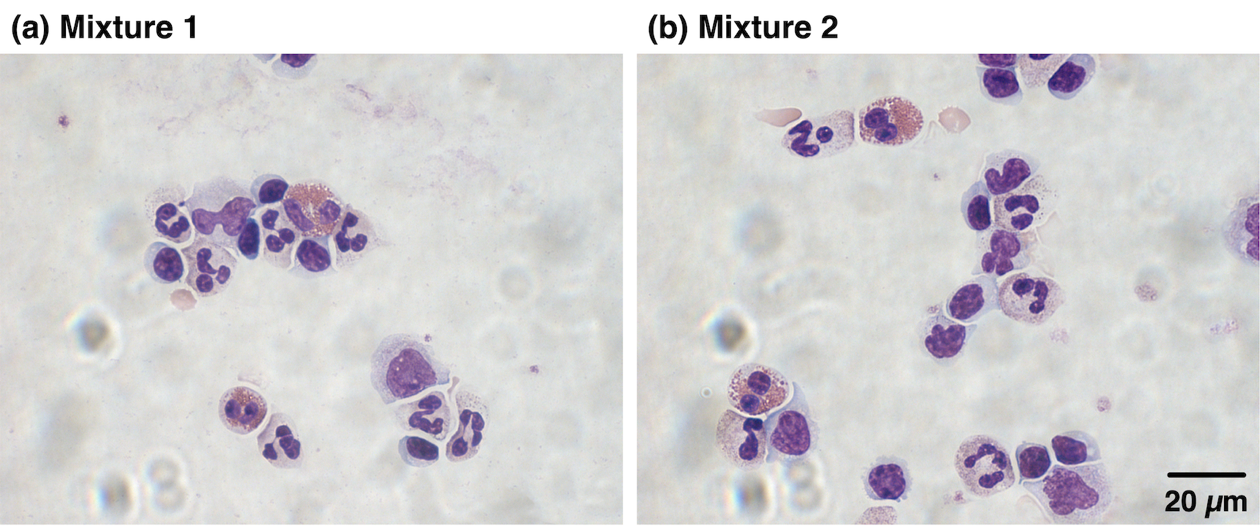


**Supplementary Figure 2.** Stained microscopic images of leukocyte mixture 1 (a) and mixture 2 (b).


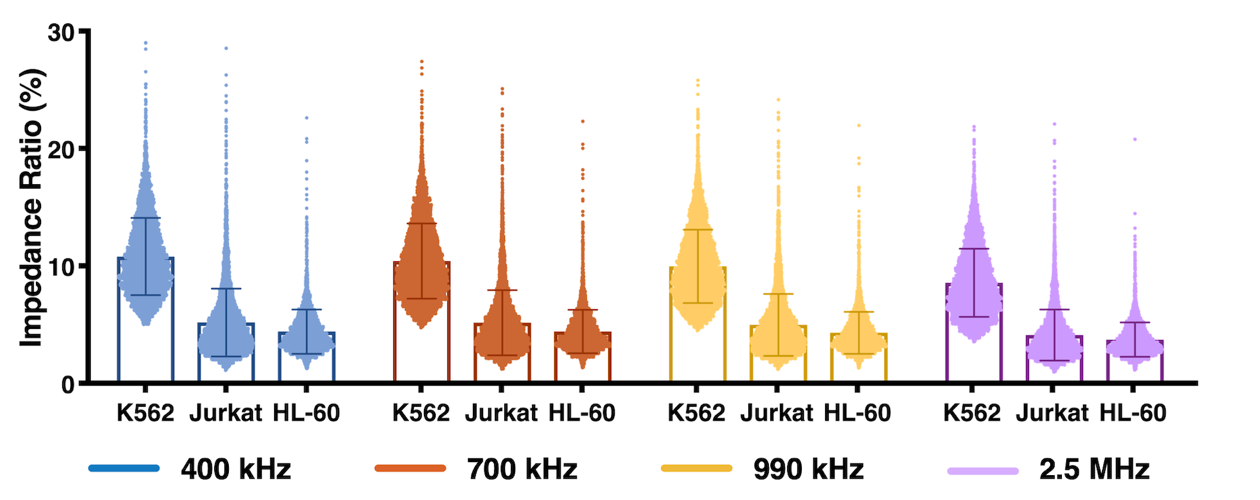


**Supplementary Figure 3.** Scatter plot of impedance ratios (amplitude with cell/amplitude without cell) for K562 (*n_cell_* = 5945), Jurkat (*n_cell_* = 6457), and HL-60 (*n_cell_* = 3524) at 400 kHz, 700 kHz, 990 kHz and 2.5 MHz.


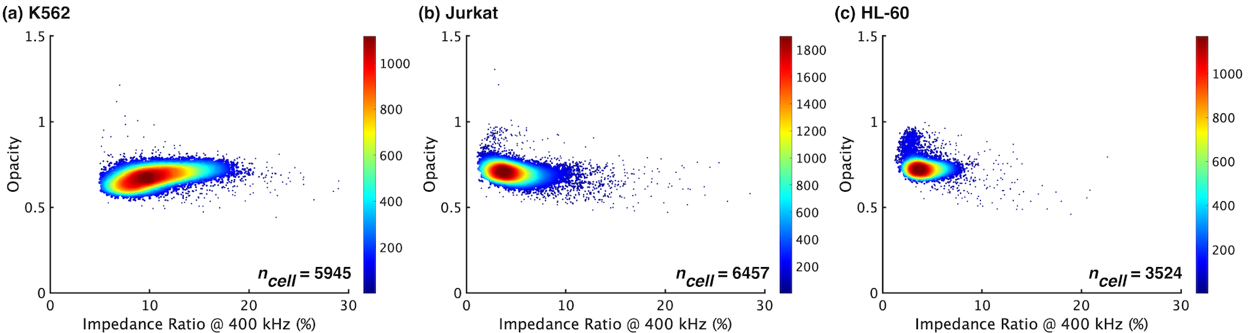


**Supplementary Figure 4.** Scatter plots of opacity (amplitude with cell at 2.5 MHz/400 kHz) *vs.* impedance ratio at 400 kHz for (a) K562 (*n_cell_* = 5945), (b) Jurkat (*n_cell_* = 6457), and (c) HL-60 (*n_cell_* = 3524).


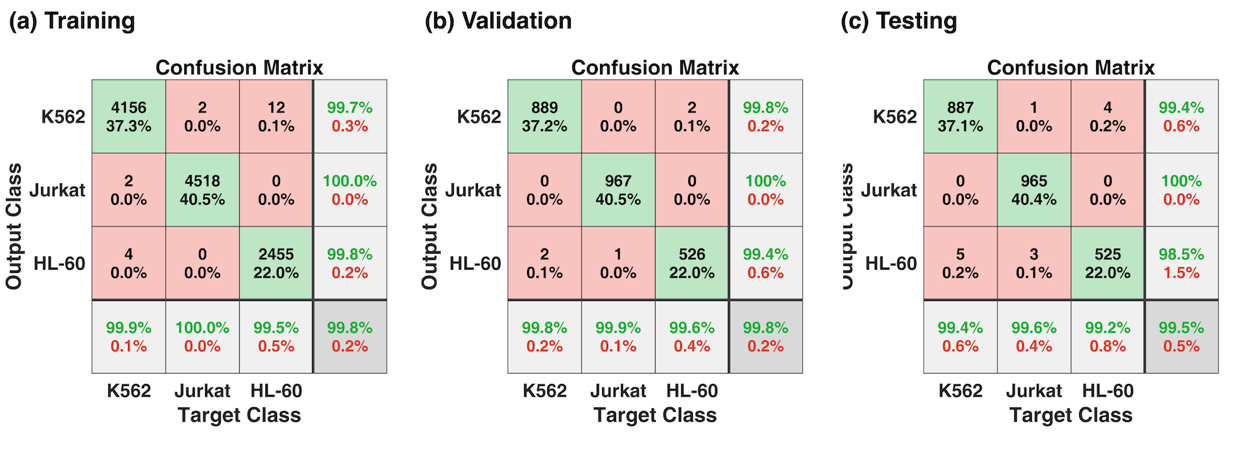


**Supplementary Figure 5.** Confusion matrices of (a) training, (b) validation, and (c) testing for distinguishing K562, Jurkat, and HL-60 cells based on single-cell impedance data collected in the virtual constriction microchannels.


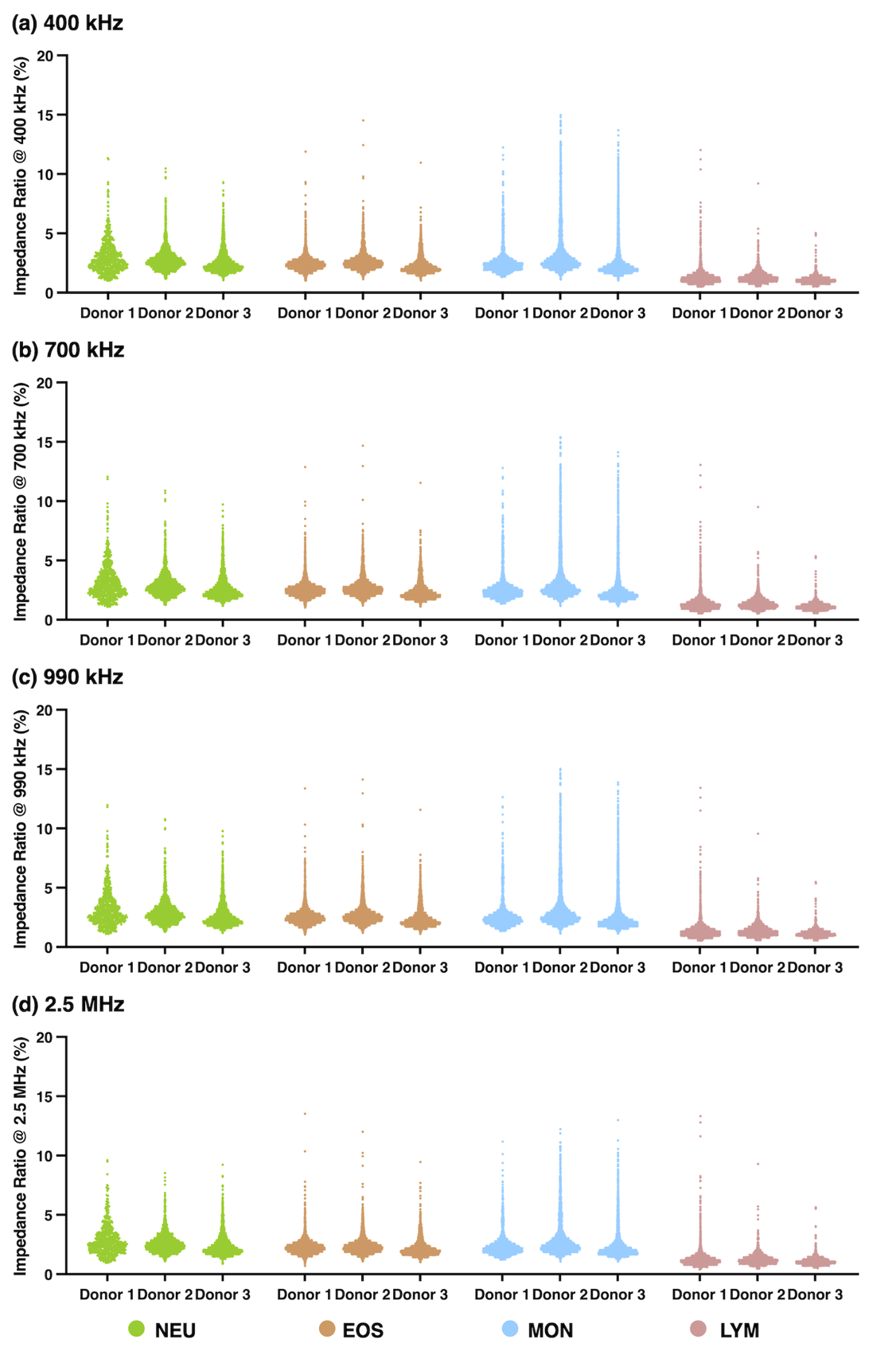


**Supplementary Figure 6.** Scatter plots of impedance ratios at (a) 400 kHz, (b) 700 kHz, (c) 990 kHz and (d) 2.5 MHz for NEU, EOS, MON, and LYM from donor 1, donor 2, and donor 3.


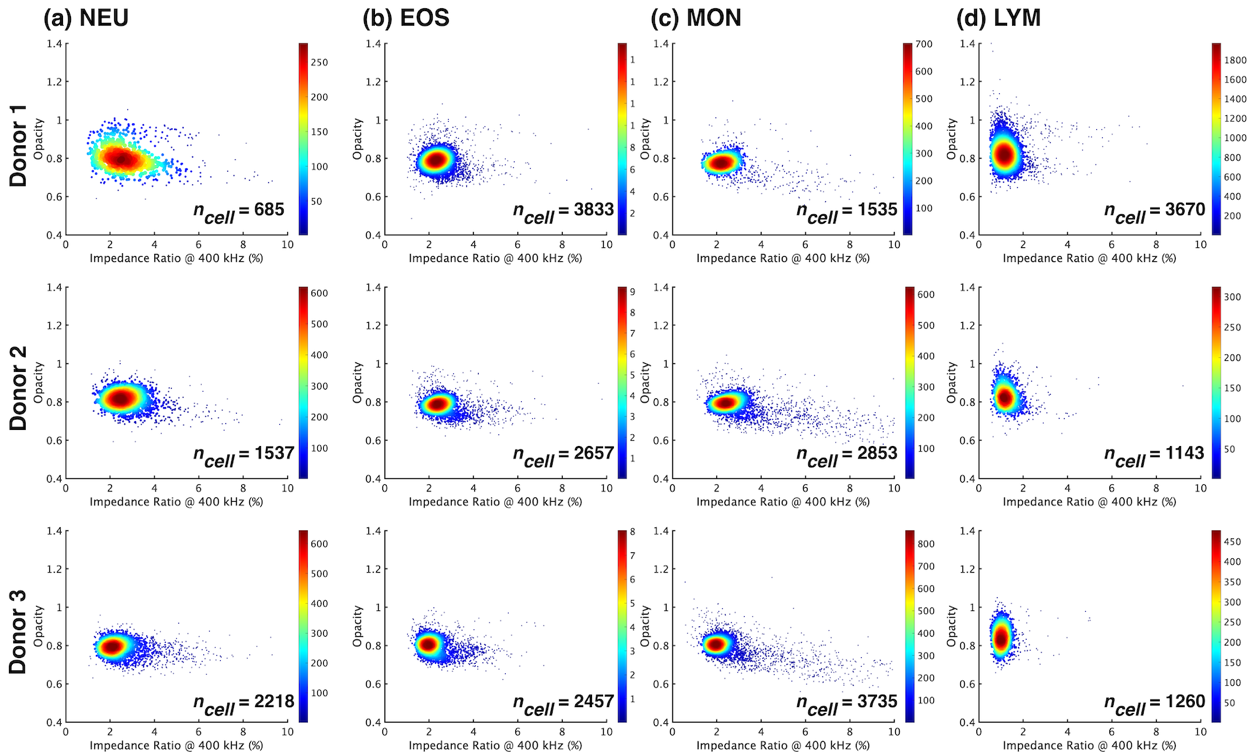


**Supplementary Figure 7.** Scatter plots of opacity *vs.* impedance ratio at 400 kHz for (a) NEU, (b) EOS, (c) MON and (d) LYM of donor 1, donor 2 and donor 3.


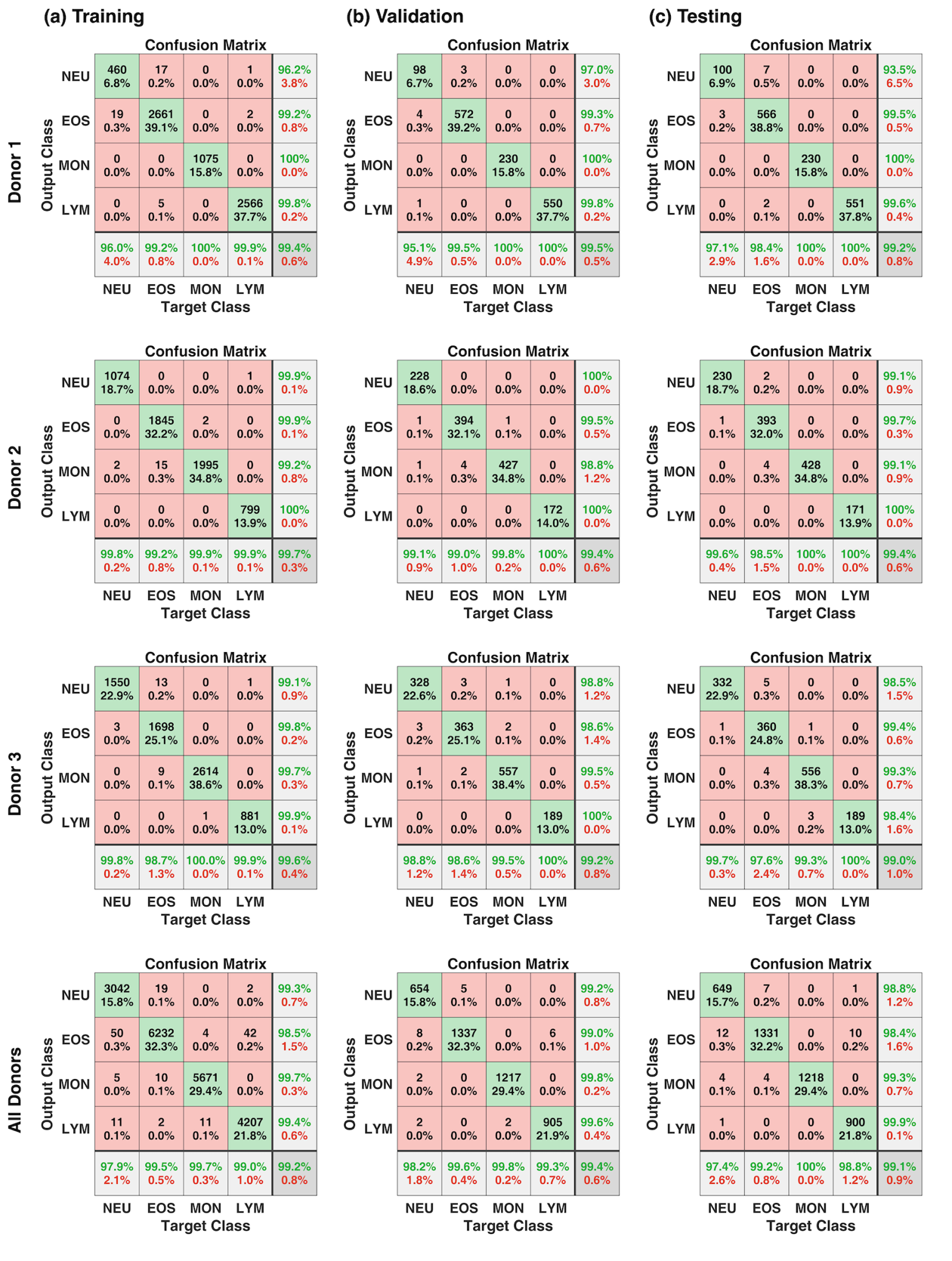


**Supplementary Figure 8.** Confusion matrices of (a) training, (b) validation, and (c) testing for distinguishing NEU, EOS, MON and LYM for donor 1, donor 2, donor 3 and all donors based on single-cell impedance data collected from the virtual constriction microchannel.


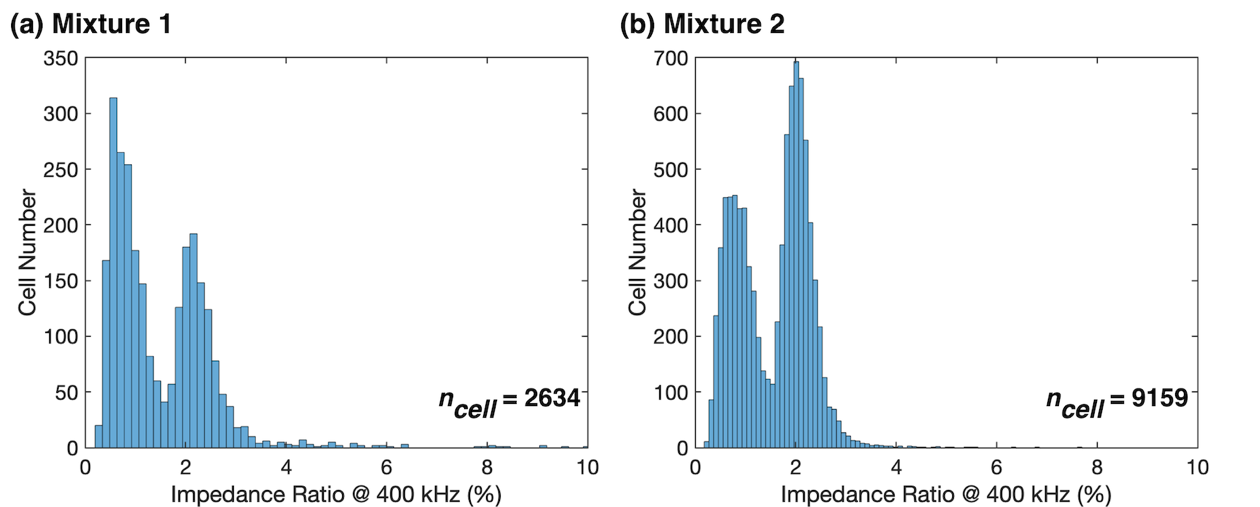


**Supplementary Figure 9.** Histograms of impedance ratios at 400 kHz for leukocyte mixture 1 (*n_cell_* = 2634) (a) and mixture 2 (*n_cell_* = 9159) (b).
